# High resolution IgH repertoire analysis reveals fetal liver as the likely origin of life-long, innate B lymphopoiesis in humans

**DOI:** 10.1016/j.clim.2017.06.005

**Published:** 2017-10

**Authors:** Anindita Roy, Vojtech Bystry, Georg Bohn, Katerina Goudevenou, Tomas Reigl, Maria Papaioannou, Adam Krejci, Sorcha O'Byrne, Aristeidis Chaidos, Andrea Grioni, Nikos Darzentas, Irene A.G. Roberts, Anastasios Karadimitris

**Affiliations:** a Department of Paediatrics, University of Oxford, Brno, Czech Republic; b CEITEC - Central European Institute of Technology, Masaryk University, Brno, Czech Republic; c Centre for Haematology, Department of Medicine, Imperial College London, Imperial College Healthcare NHS Trust, Hammersmith Hospital, London, UK; d RECAMO, Masaryk Memorial Cancer Institute, Brno, Czech Republic; e Centro Ricerca Tettamanti, Clinica Pediatrica, Università di Milano-Bicocca, Ospedale San Gerardo/Fondazione MBBM, Monza, Italy; f MRC Molecular Haematology Unit, Weatherall Institute of Molecular Medicine, University of Oxford and BRC Blood Theme, NIHR Oxford Biomedical Centre, Oxford, UK

**Keywords:** Human, Fetal, IgH repertoire

## Abstract

The ontogeny of the natural, public IgM repertoire remains incompletely explored. Here, high-resolution immunogenetic analysis of B cells from (unrelated) fetal, child, and adult samples, shows that although fetal liver (FL) and bone marrow (FBM) IgM repertoires are equally diversified, FL is the main source of IgM natural immunity during the 2nd trimester. Strikingly, 0.25% of all prenatal clonotypes, comprising 18.7% of the expressed repertoire, are shared with the postnatal samples, consistent with persisting fetal IgM + B cells being a source of natural IgM repertoire in adult life. Further, the origins of specific stereotypic IgM + B cell receptors associated with chronic lymphocytic leukemia, can be traced back to fetal B cell lymphopoiesis, suggesting that persisting fetal B cells can be subject to malignant transformation late in life. Overall, these novel data provide unique insights into the ontogeny of physiological and malignant B lymphopoiesis that spans the human lifetime.

## Highlights

• Second trimester human fetal liver and fetal bone marrow B-cells have IgM repertoires that are equally diversified
• Human fetal liver B-cells are the main source of innate, natural IgM responses
• CLL-associated, stereotypic B cell receptors are detected in fetal IgM repertoire

## Graphical abstract

**Figure:**
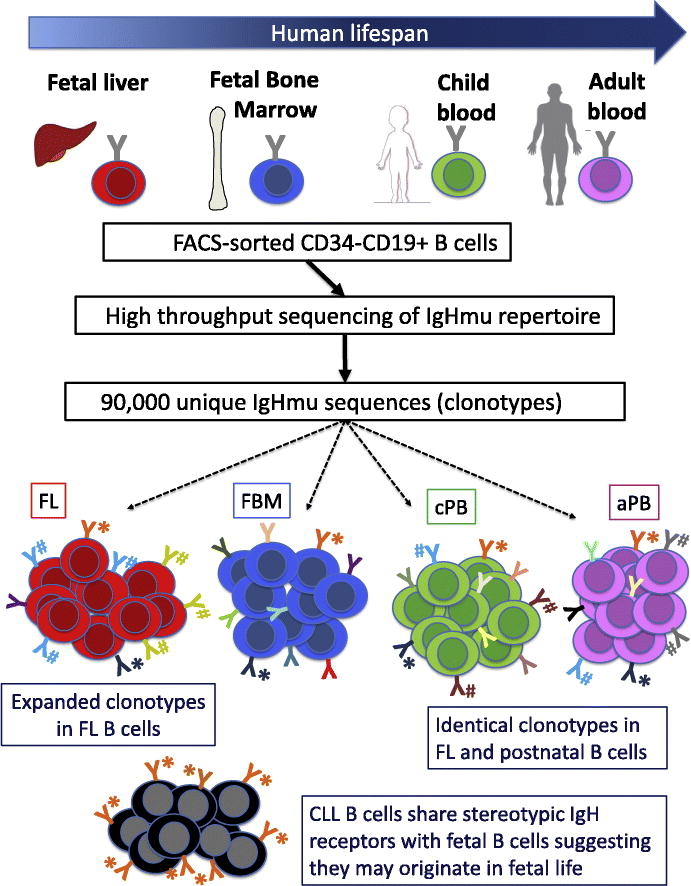

## 1 Introduction

Mature B-cell development in humans starts in the fetal liver (FL) in early fetal life, and becomes well established at this site by the start of the second trimester [1], [2]. Subsequently, during the second trimester, bone marrow (BM) becomes the main site of B lymphopoiesis [3] and remains so throughout post-natal life.

Development of mature B-cells depends upon, and proceeds commensurately with expression of a functional B-cell receptor (BCR) and of its constituent immunoglobulin (Ig) heavy(H) and light(L) chains. The molecular hallmark of B-cell development, somatic recombination of the genes that encode the IGH(V, D and J) and IGL(V and J) chains, takes place in early B-cell progenitors in primary B lymphopoiesis sites (i.e. FL, FBM and adult BM). This ensures the first wave of Ig repertoire diversification, with antigen specificity primarily encoded by the complementarity determining region 3 (CDR3). This process is a pre-requisite for efficient humoral immunity, even early in fetal life [4]. The first mature B-cells that emerge from FL and FBM are transitional B-cells that co-express IgM, IgD and CD10 [5], [6]. Transitional B-cells mature into CD10neg naïve B-cells that express less IgM. In postnatal life, but not fetal life, naïve B-cells enter a germinal centre reaction in secondary lymphoid organs, undergoing isotype class switch to IgG/IgA and somatic hypermutation, a process that ensures the second wave of Ig repertoire diversification and the production of high affinity soluble antibodies. By contrast, the majority of the fetal life IgM repertoire comprises antibodies that are self- and poly-reactive [7]. This so called ‘natural’ IgM antibody repertoire is public, i.e., shared by different individuals at birth and is present in adult life as part of the normal, non-pathogenic innate Ig repertoire, albeit at lower frequencies than in the newborn [8], [9]. Self-reactive and poly-reactive IgM antibodies, and in particular those using the IGHV6-1 gene, are dominant in FL B-cells [10]. In adult life, self-reactive IgM antibodies may play a role in protection from pathogens and autoimmunity [11]. In mice, the natural IgM repertoire is largely linked to B-1a cells which once developed and selected in FL, persist for the animal's lifespan through their ability for self-renewal rather than iterative development and selection [12]. Recent evidence suggests that B-1a-like cells also exist in humans and may contribute to the development of the natural IgM repertoire [13].

Profiling of the expressed IgH gene repertoire at mRNA level has helped to understand the dynamics of humoral immunity development. However, the relationship of the fetal B-cell IgM repertoire to post-natal child and adult B-cells is incompletely understood and has mostly been approached by low-throughput analyses [14], [15]. A recent high-throughput study of the IgH repertoire of circulating fetal blood B-cells provided some insights into Ig repertoire ontogeny [16]. However, the spatiotemporal relationship between the IgH repertoire in FL with that in FBM, and the impact of the fetal Ig repertoire on the long-term repertoire present in post-natal life, as well as the link between this and the development of disease, are unknown.

Here, to address these issues and to gain insights into the ontogeny of the human innate B-cell repertoire, we take advantage of a high-resolution analysis of the IgH-Cmu repertoire of normal human FL, FBM and post-natal B-cells from healthy infants, young children and adults.

## 2 Materials and methods

### 2.1 Samples

Human FL and BM cells (Table S1) were provided by the Human Developmental Biology Resource (www.hdbr.org). Surplus blood from samples collected from healthy children was obtained under national ethics committee approval (MREC12/LO/0425). For each sample, CD34-CD19 + mature B-cells (Table S1) were FACS sorted on BD FACSAriaII (Becton Dickinson, Oxford, UK) for BCR repertoire analysis by 454 sequencing.

### 2.2 Bioinformatics

To reduce repertoire sampling biases, we included in the analysis only samples with a comparable number of B-cells when possible (Table S1). The raw NGS data were processed, annotated with germline sequences from IMGT® and/or using IMGT/V-QUEST and IMGT/HighV-QUEST (http://www.imgt.org), and analysed through ARResT/Interrogate [17]. As part of ARResT/Interrogate, and with the use of the R language for statistical computing [www.R-project.org]: the Jensen-Shannon divergence was used to compute repertoire similarity between pairs of samples; the inverse Simpson concentration [18], which favors abundant clonotypes over rare ones, was used on vectors of clonotype abundances to calculate clonotypic diversity. Sequences were assigned to the 19 major subsets of stereotyped B-cell receptors in chronic lymphocytic leukemia (CLL) using ARResT/AssignSubsets [19].

Further methodological details are provided in Supplementary methods.

## 3 Results

### 3.1 High-resolution analysis of fetal and postnatal IgHmu repertoires

**Fig. 1:**
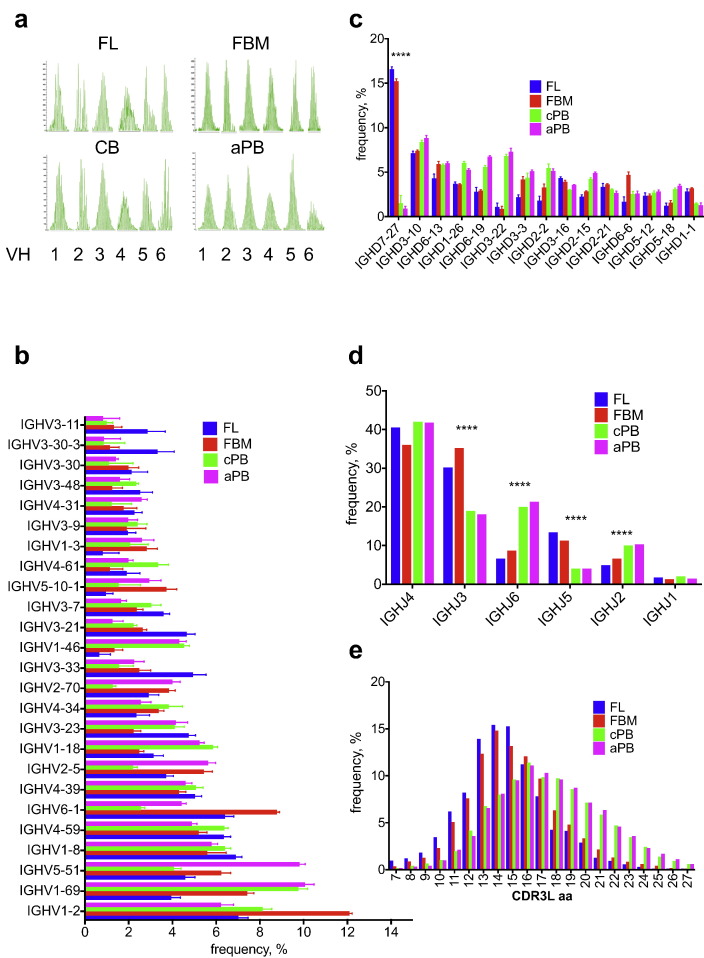
Diversification features of fetal B cell repertoire. a. Spectratyping analysis of IGHV1-6 families in fetal liver (FL) GA 15^+ 3^ weeks, fetal bone marrow (FBM) GA 15^+ 3^ weeks, cord blood (CB) and adult peripheral blood (aPB) B-cells. b. IGHV gene (the top 25 out of 52 VH genes are shown) c. IGHD gene (the top 15 out of total 25 JH genes are shown) d. IGHJ gene and e. CDR3 aa length repertoires counted in unique clonotypes in the 4 different fetal and postnatal developmental stages (cPB: child peripheral blood). b–e: mean values with SD are shown, except in d & e where error bars are omitted for simplicity. (***p < 0.001, ****p < 0.0001).

For initial assessment of the IgM repertoire ontogeny in FL and FBM B-cells, we analysed flow-sorted CD34-CD19 + B-cells. These express cytoplasmic IgM(mu) and/or surface (s)IgM and comprise pre-B-cells, immature, transitional and naïve B-cells [5], [20]. Spectratyping of IGVH-Cmu mRNA IGHV1-IGHV6 amplicons from a 2nd trimester FL sample (gestational age [GA], 15^+ 3^ weeks), a 2nd trimester FBM sample of the same GA(15^+ 3^ weeks) and B-cells from healthy children and adults revealed a polyclonal repertoire in both FL and FBM that was comparable to the postnatal samples (Fig. 1a)

To gain further insights into the ontogeny of IgH diversification, we sequenced the IGVH-Cmu mRNA IGHV1-IGHV7 family amplicons from FL, FBM and postnatal samples using next-generation sequencing (NGS) and the 454 technology. In total, 20 libraries generated from 17 individual, flow-sorted CD34-CD19 + B-cell samples were sequenced: 5 FL (4 performed in independent duplicate libraries; GA 14–18 weeks), 3 FBM (GA 13–17 weeks; different fetuses from the FL samples), 3 child peripheral blood (cPB) and 5 adult PB (aPB) B-cell samples. We obtained 117,757 unique clonotypes of which 76%(90,238) were productive, with the remainder representing unproductive rearrangements (Table S1).

Reproducibility was tested by comparing the duplicate libraries from the 4 FL B-cell samples generated and sequenced in 2 independent experiments. Principal component analysis of different combinations of immunogenetic features demonstrated clear demarcation and tight clustering of duplicate pairs (Fig. S1), showing the high degree of accuracy and reproducibility of the assay.

### 3.2 Diversification of the fetal IGHV, IGHD and IGHJ repertoires

Further dissection of the complexity of IgM repertoire development showed that all 52 member genes of the IGHV1-IGHV7 families were used at varying and often significantly different frequencies in all 4 developmental stages (Fig. 1b and Table S2). In line with previous reports [14], [15], [16], the most notable difference in IGHD genes usage frequency was the > 10 fold higher IGHD7-27 frequency in fetal compared to postnatal samples (Fig. 1c). The pattern of IGHJ repertoire usage was nearly identical between FL and FBM, and between cPB and aPB B-cells. IGHJ4 was the most frequently used J gene in all developmental stages (Fig. 1d) and, consistent with previous reports [15], [16], there was reciprocal presence of 4 IGHJ genes: IGHJ6 and IGHJ2 were significantly over-represented in postnatal B-cells (*p* < 0.001), while IGHJ3 and IGHJ5 were significantly over-represented in fetal B-cells (*p* < 0.001; Fig. 1d). Finally, as previously described [15], [16], average CDR3 length was significantly shorter in fetal than postnatal B-cells, 14.8 amino acids (aa) vs. 17.3aa (*p* = 0.001; Fig. 1e)

These data show the molecular mechanisms responsible for VDJ recombination-dependent repertoire diversification are active and efficient early in B-cell development in both FL and FBM and that on a qualitative level, comparably diversified B-cell lymphopoiesis exists contemporaneously in FL and FBM.

### 3.3 Evidence of antigen-driven clonotypic expansions in FL B-cells

**Fig. 2:**
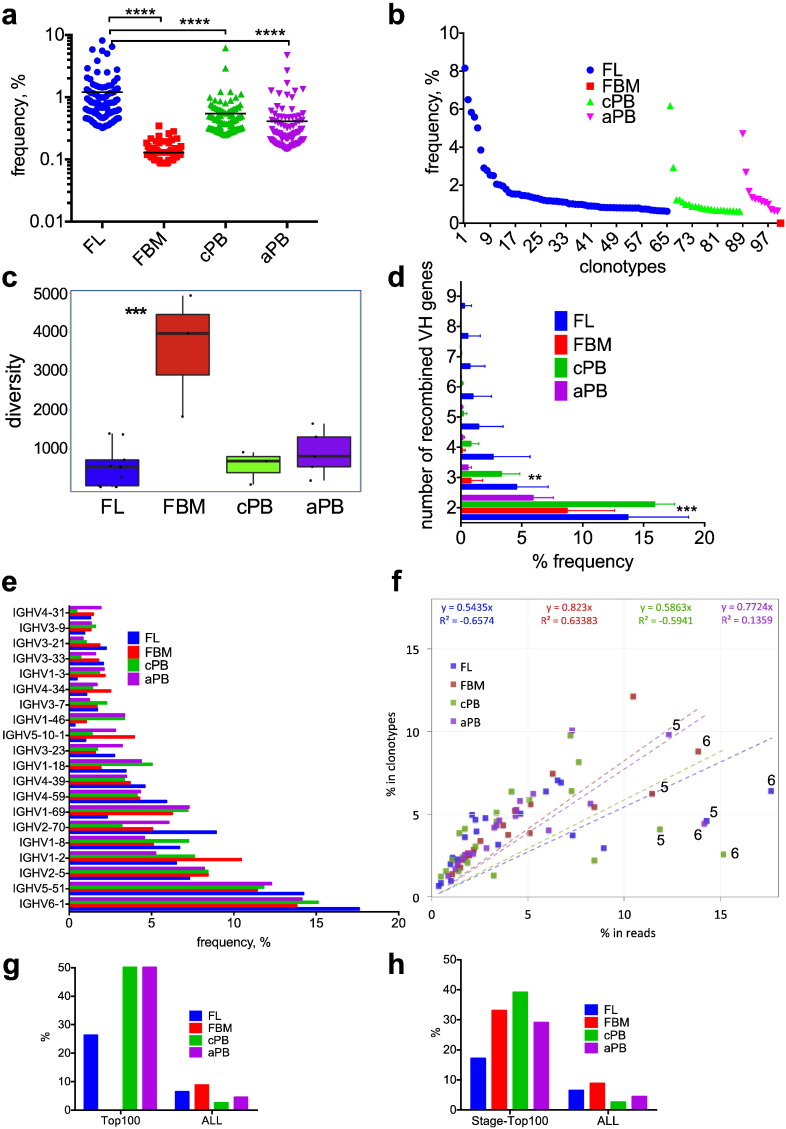
Clonotypic abundance and diversity in fetal and post-natal B-cells. a. Frequency (abundance counted in reads) of the 100 most abundant clonotypes in each developmental stage (horizontal lines indicate mean values, ****p < 0.0001). b. Distribution of the 100 most abundant clonotypes across the 4 developmental stages. c. Clonotypic diversity in each developmental stage as assessed by the inverse Simpson concentration (see Methods). ***p < 0.001 for FBM as the most diverse. d. Frequency of CDR3 peptides generated by convergent recombination of 2–9 different IGHV genes in each developmental stage. ** and *** for *p* < 0.01 and *p* < 0.001 respectively for FL and cPB vs. FBM and aPB. e. IGHV gene repertoire counted in reads in fetal and postnatal B-cells. Mean values are shown (error bars omitted for simplicity). f. Correlation of the relative clonotype abundances of the 20 most popular IGHV genes across developmental stages with their corresponding relative read counts. Lower slopes (as indicated by ‘y’ values in respective colors) of the regression lines for FL and cPB indicate the predominance of high abundance clonotypes in these developmental stages. ‘5’ and ‘6’: outlying and highly expressed IGHV5-51 and IGHV6-1 respectively. g & h. IGHV6-1 usage in the 100 most abundant clonotypes across developmental stages (g), and in the 100 most abundant clonotypes in each developmental stage (h); these “Top100” frequencies are compared to those of IGHV6-1 usage in all clonotypes (“ALL”).

Antibodies produced by the fetus are mostly IgM and are self- and poly-reactive; however, the source of fetal IgM in FL or FBM B-cells is not known. Hypothesising that B-cells producing IgM would have undergone clonotypic expansion in response to self-antigenic stimulus, we sought to identify such expansions by studying the 100 most abundant clonotypes in each stage. Mean clonotype abundance of the top 100 most abundant clonotypes in each of the 4 stages, was 10-fold lower in FBM B-cells (0.12%) than in FL B-cells (1.2%, *p* < 0.0001), while corresponding abundances in postnatal PB B-cells were intermediate (cPB: 0.54%; aPB: 0.41%; Fig. 2a). Reflecting the paucity of expanded clonotypes amongst FBM B-cells, analysis of the 100 most abundant clonotypes from across all 4 stages (i.e., 100 of 90,238 clonotypes, Table S1) showed that none were present in FBM, compared to 65 in FL, 23 in cPB, and 12 in aPB B-cells (Fig. 2b)

To assess clonotypic expansion and diversity in individual stages, we estimated the inverse Simpson concentration of the clonotypic repertoires [18]. We found that FL clonotypes are the least diverse, followed by cPB and aPB, while FBM showed significantly higher clonotypic diversity compared to the other groups (*p* < 0.001; Fig. 2c).

Together these results are consistent with robust IgM B-cell clonotypic expansions being prominent in FL and nearly absent in FBM of the same GA, and support the notion that the FL B-cells are the main source of fetal IgM production during the 2nd trimester.

### 3.4 Convergent recombination in fetal B-cells

We sought further evidence of antigen-driven responses amongst fetal B-cells by studying their clonotypes in detail, focusing first on clonotypes shared between the duplicate FL libraries to mitigate against possible PCR/sequencing artefacts. All 4 duplicate FL libraries showed evidence of distinct VDJ rearrangements encoding identical CDR3 peptide regions, involving 95–185 clonotypes (1.9–9.3% of all unique clonotypes per library; Table S3). Notably, these CDR3 regions were identical both at the aa and nucleotide level using either different IGHV or IGHD genes, but always the same IGHJ gene (Tables S4 & S5). This strikingly precise selection of CDR3 regions, previously termed convergent recombination, has been described for T-cell receptor repertoire [21], [22] and recently in murine B-1a cells [23]. We investigated this further by systematically searching for multiple IGHV genes recombined to an identical CDR3 aa sequence in all samples. Across all stages we found evidence of hundreds of CDR3 sequences recombined with 2–4 different IGHV genes, with up to 9 different IGHV genes identified (Fig. 2d); in nearly all cases this involved genes of the same IGHV family. Detailed sequence analysis (Table S5) highlights the unambiguous assignment of respective germline sequences with no signs of PCR hybrids. Importantly, CDR3 sequences involved in convergent recombinations were most abundant in FL and cPB (Fig. 2d) in line with their increased incidence of prominent clonotypic expansions (Fig. 2a–c)

Therefore, convergent recombination, a process that ensures generation of a high abundance public immune repertoire in T-cells [21], [24], also appears to shape the early fetal B-cell repertoire.

### 3.5 Abundant IGHV6-1 repertoire across developmental stages

To investigate whether repertoire complexity is influenced by biases in specific IGHV family member usage, we compared the rankings of IGHV genes by their frequency of unique clonotypes (i.e. counting in unique clonotypes; Fig. 1b) and abundance (i.e. counting in sequence reads; Fig. 2e). IGHV6-1 and IGHV5-51 were the 1st and 2nd most expressed genes across all 4 developmental stages; however, both genes ranked lower when counted in unique clonotypes, especially in postnatal samples. Fig. 2f correlates relative clonotype abundances of the 20 most popular IGHV genes across developmental stages with their corresponding relative read counts. Expecting these two measures to be linearly correlated, outliers should highlight IGHV genes with highly/lowly-expressed clonotypes. We found that in all 4 developments stages, IGHV6-1 and IGHV5-51 are placed the furthest from their projected linear distribution and strongly biased towards high expression. Therefore, although IGHV6-1 and IGHV5-51 genes did not have the most associated clonotypes, they are the most likely to participate in high abundance, expanded clonotypes. At a global level, the lower slopes of the regression lines for FL and cPB are also consistent with the higher frequency of high abundance clonotypes in those samples

We then focused on IGHV6-1 as this has previously been shown to be over-represented in fetal B-cells beyond its expected frequency of ~ 1.9% (i.e., 1/52) [25], [26], [27]. Fetal IGHV6-1 IgM BCRs have been reported to react against ssDNA and cardiolipin autoantigens, and are thus important sources of natural IgM [10]. We confirmed that although the relative frequency of IGHV6-1 in unique clonotypes was not higher than the expected 1.9% in FL and FBM (Fig. 1b), its abundance was indeed significantly higher (18% and 14% respectively; *p* = 0.002; Fig. 2e) with similar trends in cPB and aPB. Supporting this, IGHV6-1 was identified in 17/65(26.2%) FL, 11/23(50%) cPB and 6/12(50%) aPB of the 100 most expanded clonotypes across all 4 stages (Fig. 2g). Similarly, within each developmental stage, IGHV6-1 comprised 17, 33, 39 and 29 of the 100 most abundant clonotypes respectively (Fig. 2h), significantly higher frequencies (*p* < 0.01) than their respective average unique clonotype frequencies (6.4, 8.7, 2.5 and 4.4%). Thus, IGHV6-1 clonotypic expansions are dominant in all developmental stages, highlighting an important role of IGHV6-1 IgM in innate humoral immunity throughout life.

### 3.6 Presence of antigen response-competent mature B-cells in FL but not FBM

**Fig. 3:**
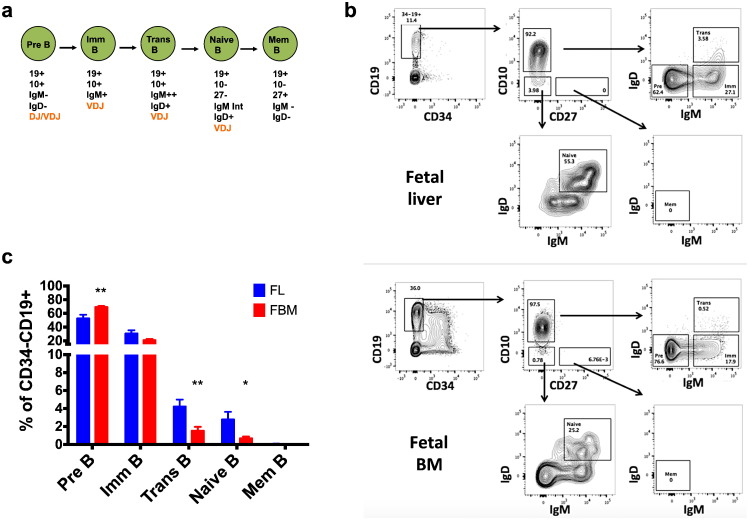
B cell development in FL and FBM. a. Schematic representation of proposed fetal B cell maturation according to immunophenotypic markers and stages of VDJ recombination (using human fetal B cell development data where available) [5], [6], [29] that was studied in 2nd trimester FL and FBM. b. Representative flow-cytometric analysis of FL and FBM of the same fetus (GA 17 weeks) showing the gating strategy used to identify the various stages of B cell maturation as described in (a). Data are from viable CD34 negative cells for the FL sample and viable mononuclear cells for the FBM sample. c. Frequencies of the B cell stages, expressed as % of CD34-CD19 + cells, are shown in the bar graph with data represented as mean ± SEM from FL (*n* = 13) and FBM (*n* = 12) samples. (Imm: immature, Trans: transitional, Mem: memory B-cells; *p < 0.05, **p < 0.01).

The high frequency of expanded clonotypes in FL but not FBM suggests that in fetal life it is the FL rather than FBM B-cells that mount (auto-)antigen-driven responses, despite being equally diversified by VDJ recombination. To investigate this further, we compared the frequencies of B-cell sub-populations within the CD34-CD19 + compartment in FL and FBM using previously described markers especially those defining fetal B cell subsets where available [5], [6], [20], [28], [29] (see Supplementary methods). While pre-B-cells lack (s)IgM expression, immature B-cells, transitional and naïve B-cells express (s)IgM (Fig. 3a, b). Compared to FL, the FBM CD34-CD19 + compartment had a higher frequency of pre-B-cells (FL: 52.7 ± 5.4% vs. FBM: 69.2 ± 1.5%, *p* < 0.01) but a similar frequency of immature B-cells (30.7 ± 4.6% vs. 21.0 ± 1.6%), while transitional and naïve B-cells were significantly decreased in FBM (FL: 4.2 ± 0.8% vs. FBM: 1.5 ± 0.4%, *p* < 0.01; and FL: 2.8 ± 0.9% vs. FBM: 0.7 ± 0.2%, *p* < 0.05; Fig. 3c). This lack of developed mature B-cells explains, at least in part, the paucity of clonotypic expansions in 2nd trimester FBM. Of the three sIgM + B-cell populations (immature, transitional and naïve) we used for IgHmu repertoire profiling, only the transitional B-cell subset was previously shown to expand in response to antigen in a T-cell-independent fashion. Indeed, transitional B-cells are enriched in autoreactive B-cells in normal individuals and more so in patients with systemic lupus erythematosus [30]. Thus, we speculate that transitional B-cells are likely to be the main source of the FL IgM clonotypic expansions and, in contrast to previous reports [4], [31], we found a very low frequency of CD34-CD19 + CD27 + B-cells in 2nd trimester FL and FBM (range 0–1.9% of total CD34-CD19 + B-cells, median 0.06%). (Fig. 3b & c).

### 3.7 High abundance FL clonotypes shared across developmental stages

**Fig. 4:**
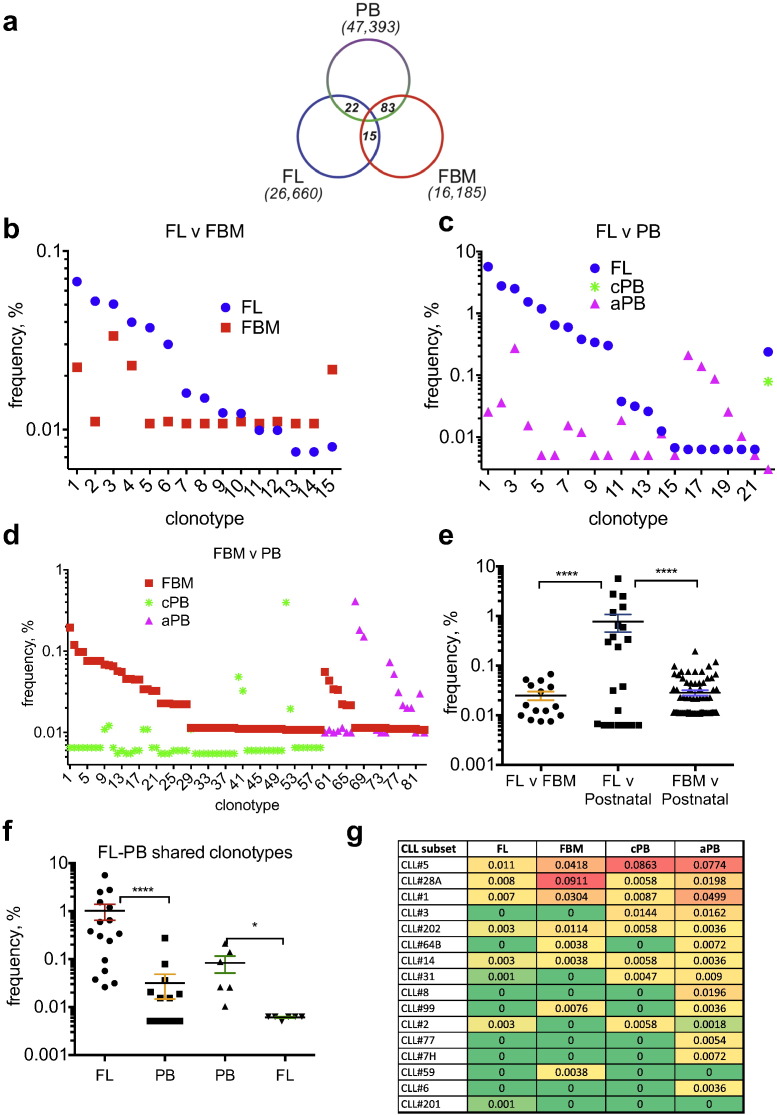
Sharing of clonotypes across developmental stages. a. Venn diagram showing the distribution of the 120 shared clonotypes between FL, FBM and post-natal PB cells. b, c & d. Sharing of clonotypes between FL and FBM, FL and post-natal, and FBM and post-natal B-cells, respectively. None of the clonotypes was shared by > 2 developmental stages. Details of the clonotypes shown in b, c & d are shown in Tables S6, S7 & S8 respectively. e. Abundance of clonotypes shared between FL and FBM, FL and post-natal, and FBM and postnatal B-cells. f. Abundance of FL-PB shared clonotypes. Two left columns show shared clonotypes whose abundance is higher in FL rather than in PB, the two right-most columns show shared clonotypes with abundance higher in PB rather than in FL. g. Abundance (%) and sharing of stereotypic IgH receptors associated with CLL in fetal and postnatal B-cells showing 11 subsets shared between FL and FBM, 13 subsets shared between child and adult PB B-cells and 3 subsets shared across all 4 stages (see Table S9; *p < 0.05, ****p < 0.0001).

To explore continuity in IgM B-cell immunity between fetal and adult life, we searched for clonotypes shared within and between developmental stages. Overall 0.13% (122/90,238) of productive clonotypes were shared, with none shared by > 2 developmental stages (Fig. 4a); 15 were shared between FL and FBM (expressed as 0.37% and 0.22% of reads respectively) (Fig. 4b and Table S6), suggesting selection of B-cells by the same antigen can occur independently in FL or FBM, or possibly migration of B-cells between sites; 2 clonotypes were shared between cPB and aPB B-cells (not shown); 22 of the total FL IgH expressed repertoire (Table S7) were shared between FL (16.3% of reads) and postnatal B-cells (0.92% of reads; Fig. 4c); and 83 were shared between FBM (2.3% of reads) and either cPB (59; 0.85% of reads) or aPB (24; 1.13% of reads; Fig. 4d, Table S8).

Reflecting the high abundance clonotypes in FL, the mean abundance of clonotypes shared between FL and postnatal B-cells was 38-fold higher than FBM (0.77% vs. 0.02%, *p* = 0.001; Fig. 4e), highlighting sharing of only high abundance clonotypes between FL and postnatal B-cells (Fig. 4e). Indeed, 10/22 clonotypes shared between FL and postnatal B-cells were also amongst the 100 most abundant clonotypes across all developmental stages (Fig. 2b, Table S7) and 16/22 shared clonotypes were 41-fold more abundant in FL B-cells than in postnatal B-cells (median 0.36% vs. 0.005%, *p* < 0.0001; Fig. 4f). Notably, 5/22 FL-postnatal shared clonotypes, corresponding to 2 individual CDR3 sequences, had evidence of convergent recombination (Table S7), supporting the notion that clonotypic expansions shared between fetal and post-natal IgM B-cell repertoires are antigen-driven.

Together, these observations are consistent with a fully functional FL IgM repertoire in which B-cell clonotypic expansions are robust and likely to be antigen-driven. The presence of identical clonotypes in fetal and postnatal B-cells might be the result of independent selection at different developmental stages in different individuals or, more likely, selection during fetal life and subsequent persistence in postnatal life. Further, the higher abundance of some shared clonotypes in postnatal compared to FL B-cells indicates that IgM-producing B-cells of FL origin remain functional in postnatal life and retain their ability to expand in response to recurrent antigenic stimulation. Finally, the unexpected degree of clonotype sharing (0.25% of the entire fetal IgM repertoire) between fetal and postnatal B-cells derived from samples that are HLA-disparate suggests that the selection of these shared (public) clonotypes occurs in an HLA- and thus T-cell-independent manner consistent with IgM innate humoral immune responses.

### 3.8 Fetal BCR repertoire and malignancy-associated stereotypic receptors

Our results so far suggest that fetal IgM-producing B-cells may persist into adult life and remain under antigenic stimulation throughout life, potentially increasing their risk of neoplastic transformation. Stereotypic (or quasi-identical) IgM BCR are known to be part of the normal adult B-cell repertoire (enriched in IgM + CD5 + B-cells in particular [32]) and, importantly, they have also been demonstrated in ~ 30% of patients with chronic lymphocytic leukemia (CLL), one of the most common IgM + mature B cell malignancies in humans [33], [34], [35], [36]. Nevertheless, their developmental origins and ontogeny have not been defined. We therefore searched for evidence in our fetal and postnatal IgM-producing B-cell samples, for the 19 major stereotypic CLL IgH receptors, or major CLL subsets, previously reported in a large study of > 7500 CLL patients [33]. Overall, we found evidence of stereotypic IgH receptors corresponding to one or more of the 16 major CLL subsets in 3/5 FL B-cell samples and in all FBM and postnatal samples (Fig. 4g and not shown): 14/16 subsets were found in postnatal samples, with CLL#1, CLL#5, and CLL#28A the most prevalent but strikingly, 11/16 stereotypical subsets were also present in fetal B-cells, with 2 subsets in fetal B-cells only (Fig. 4g, Table S9). CLL#1, CLL#5 and CLL#28A subsets were again the most prevalent in FL and FBM B-cells (Fig. 4g). Importantly CLL#1, CLL#5 and CLL#28A are amongst the 10 most common CLL subsets, and CLL#1 and #5 are associated with aggressive disease [36]. These findings may provide clues into the ontogenesis of CLL and indicate that for a substantial proportion of stereotypy-associated CLL, the IgM + B-cell that undergoes malignant transformation in adult life may originally be selected during fetal life and persist throughout adulthood.

## 4 Discussion

Here we present a comparative, high-resolution dissection of the human IgHmu repertoire from early fetal to adult life. This is the first such analysis to include FBM and FL, the primary sites of fetal B-cell development thus allowing ontogenic and anatomical mapping of the human natural IgM repertoire.

The IgHmu repertoire in prenatal life is responsible for development of the so-called natural antibody immunity. Work in mice has shown that development of B-cells secreting natural IgM is instructed by non-protein, lipid, phospholipid and glycan antigens often from cells undergoing apoptosis [37], [38], [39]. In this respect natural IgM are low affinity auto-reactive antibodies perhaps triggered by inadequately cleared apoptotic cells during fetal development [40]. In postnatal life, the natural IgM repertoire is further enriched with specificities against commensal flora or pathogen-derived non-protein antigens [40]. In mice, the main cellular source of natural IgM are B-1a cells that develop in FL but not FBM. After their selection and clonal expansion by auto-antigens, they persist throughout life by self-renewal.

Our analysis of the ontogeny of the corresponding human IgHmu repertoire, not previously characterised, reveals many features analogous to mice. We find that while comparably diversified B-cell lymphopoiesis exists contemporaneously in FL and FBM, the robust IgM-producing B-cell clonotypic expansions prominent in FL are virtually absent in FBM of the same GA, thus identifying human FL as the likely main source of the natural IgM repertoire in fetal life. The lack of clonotypic expansions in FBM reflects the paucity of late mature B-cells; these probably develop in late 3rd trimester to become the main source of adaptive B-cells in postnatal life. Given that the cord blood and early neonatal IgM repertoires are functionally autoreactive, these clonotypic expansions are likely to be auto-antigen-driven. Indeed, IGHV6-1 clonotypic expansions were dominant in FL, and human IGHV6-1 + fetal B-cells have previously been shown to be reactive against self-phospholipids such as cardiolipin [10]. The corresponding orthologous VH7183.1 is also dominant in murine FL [41] revealing remarkable and refined evolutionary conservation.

As in mice, the human natural IgM repertoire is also public, comprising identical/near-identical clonotypes shared by different individuals. Our data provide the first evidence of considerable sharing of IgM clonotypes that originate in human FL amongst different fetuses. For some FL clonotypes, we documented stringent (even at the nucleotide level) convergent recombination underpinning public IgM repertoire generation. While convergent recombination occurring at the aa level has been described in the adult Ig repertoire [42], [43], [44], nucleotide-level convergent recombination was recently described in murine B1 cells [23], providing further parallels between human and murine natural IgM ontogeny.

Another distinct contribution of our work to the delineation of the ontogeny of the postnatal ‘public’ IgM repertoire is the finding that FL expanded clonotypes, including those with IGHV6-1, are also found clonally expanded in postnatal life. This most likely reflects auto-reactive IgM-producing B-cells, clonotypically expanded in FL, persisting throughout life perhaps bypassing FBM. Whether their postnatal persistence and expansion is the result of continuous antigenic stimulation (e.g., by apoptotic cells) or of their ability to self-new (analogous to murine B-1a cells) remains to be determined.

Recent IgH repertoire analysis of fetal B cell progenitors at a single cell level, demonstrated that the distinct immunogenetic features of fetal IgH repertoire are determined, at least in part, by a fetal-specific pattern of VDJ recombination process which may be driven by differences in Tdt expression in fetal life [29].

While analysis of the human fetal IgM repertoire has revealed high concordance with the corresponding murine repertoire, our analysis of the cellular correlate of the pre-immune repertoire, i.e., of B-1a cells, has not. The existence of human counterparts of murine B-1a cells has been contentious. Recent work identified a rare IgM + CD20 + CD27 + CD43 + B-cell population in human cord blood and PB with several functional features akin to murine B-1a cells [13], [31]. However, despite strict gating and use of two different anti-CD27 mAb clones (data not shown), the frequency of CD19 + CD27 + cells in the FL and FBM samples we analysed was consistently < 1% with most samples having no CD19 + CD27 + cells (Fig. 3). Instead, we found FL but not FBM enriched in immunophenotypically-defined transitional B-cells, a population that in humans has also been linked with production of autoreactive IgM, autoimmune disease and a CD27-CD5 + phenotype [30], [45]. This raises the possibility that in humans the B-cell subset responsible for FL IgM clonotypic expansions has features that at least in part overlap with transitional B-cells. In future work, functional characterization and high resolution analysis of the IgHmu repertoire in purified FL and FBM B-cell subsets (Fig. 3) would be required to address this question.

Another novel insight from our work is the demonstration that stereotypic, autoreactive BCR with innate function that are associated with CLL, a malignancy of older adults, may be selected during fetal life. We find that the frequencies of these stereotypic receptors in both FL and FBM are overall very low (< 0.01%; Fig. 4g) and nowhere near the frequencies of expanded clonotypes in FL (up to 8%; Fig. 2b) implying that auto-antigens driving their expansion in late adult life may not present in fetal life.

Notwithstanding the very low frequency of CD27 + mature B-cells we observed in FL and FBM, this would support the notion that CD5 + B-cell CLL with unmutated BCR might have its origin in FL B-1a-like B-cells, which also express CD5 and although they are selected once during fetal life they persist long-term in postnatal life [46], [47]. Alternatively, and more consistent with our immunophenotyping findings (Fig. 3), unmutated CLL has been mooted to originate from autoreactive transitional IgM + CD5 + B-cells [48]. Previous gene expression profiling of PB human B-cells identified CD27-CD5 + cells as the likely physiologic counterpart of the unmutated CLL B-cells [32]. We speculate that ongoing and life-long antigenic stimulation of these innate B-cells with stereotypic BCR originating in FL renders them susceptible to malignant transformation resulting in CLL.

In conclusion, comparative analysis of IgM repertoire development from fetal to adult IgM B-cells reveals that B-cell repertoire diversification during the 2nd trimester takes place in parallel in FL and FBM. However, since we have shown that mature B-cells capable of antigenic responses are present in FL but not in FBM, this suggests that the liver is the dominant site of likely self-antigen-driven B-cell clonotypic expansions during the 2nd trimester of fetal life. Such FL-derived expanded IgM + B-cells, including those of the IGHV6-1 gene, may persist into adult life and contribute to the auto- and poly-reactive public IgM repertoire and even become targets of malignant transformation.

The following are the supplementary data related to this article.

Supplementary tables Image 2

Fig. S1 Fig. S1

Supplementary methods. Image 3

## Authorship contributions

I.R and A.K designed and supervised the study. A.R, G.B, K.G, M.P, S.OB and A.C performed the experiments and did the data collection. V.B, T.R, A.K, A.G and N.D did the data analysis and interpretation of statistical data. A.R, I.R, N.D and A.K. wrote the paper and created the figures. All authors reviewed the drafts of the paper and gave final approval of the version to be published.

## Conflict of interest

The authors declare no conflict of interest.
