# Supplementary material for: High resolution IgH repertoire analysis reveals fetal liver as the likely origin of life-long, innate B lymphopoiesis in humans
Source: Clin Immunol. 2017 Oct;183:8–16. doi: 10.1016/j.clim.2017.06.005 (PMC5678457; doi:10.1016/j.clim.2017.06.005)
Supplement: Supplementary file 1 — Supplementary tables [file mmc1.pdf]

Supplementary Table 1. Samples studied and their NGS output

	sample	gestation age	No of cells	total reads count	unique clonotypes	productive clonotypes per replicate	productive clonotypes per sample
1	FL56-rep1	16wks	3000	13870	6643	5243	7854
2	FL56-rep2	16wks	3000	8088	5244	4011	
3	FL66-rep1	14wks	3000	12551	2066	1275	1882
4	FL66-rep2	14wks	3000	7956	1778	1032	
5	FL76-rep1	18wks	3000	13492	7088	5626	8588
6	FL76-rep2	18wks	3000	8181	5635	4408	
7	FL86-rep1	14wks	3000	15046	6200	4753	7045
8	FL86-rep2	14wks	3000	8783	4882	3653	
9	FL36	16wks	500	16023	2080	1291	1291
10	FBM77	17wks	1500	9047	6988	5673	5673
11	FBM87	19wks	1500	8908	7212	5886	5886
12	FBM97	13wks	3000	9271	5749	4626	4626
13	cPB97	4mo	4000	15442	7060	5703	5703
14	cPB77	4yrs	4000	16694	7983	6526	6526
15	cPB87	4yrs	4000	18690	4374	3193	3193
16	aPB37	29yrs	4000	9645	6144	5006	5006
17	aPB47	43yrs	4000	10134	7672	6451	6451
18	aPB57	40yrs	4000	9364	6401	5234	5234
19	aPB67	38yrs	4000	8824	4623	5703	5703
20	aPB66	53yrs	4000	19632	11753	9577	9577
	**Total**			**239641**	**117575**	**94870**	**90238**

Supplementary Table 2. Significance of frequency variability usage of VH genes amongst unique clonotypes according to developmentlal stage

	aPB vs. cPB	aPB vs. FBM	aPB vs. FL	cPB vs. FBM	cPB vs. FL	FBM vs. FL
IGHV4-34	**	ns	ns	ns	***	*
IGHV2-70	****	ns	**	****	****	*
IGHV1-8	ns	ns	**	ns	ns	**
IGHV4-59	***	ns	****	*	ns	**
IGHV3-7	***	ns	****	ns	ns	**
IGHV3-48	ns	ns	ns	*	ns	**
IGHV1-2	****	****	*	****	**	****
IGHV1-69	ns	****	****	****	****	****
IGHV5-51	****	****	****	****	ns	****
IGHV6-1	****	****	****	****	****	****
IGHV2-5	****	ns	****	****	****	****
IGHV3-23	ns	****	ns	****	ns	****
IGHV3-33	ns	ns	****	ns	****	****
IGHV3-21	*	***	****	ns	****	****
IGHV5-10-1	***	ns	****	****	ns	****
IGHV1-3	ns	ns	****	ns	**	****
IGHV3-30-3	ns	ns	****	ns	****	****
IGHV3-11	ns	ns	****	ns	****	****
IGHV4-39	ns	ns	ns	ns	ns	ns
IGHV1-18	ns	****	****	****	****	ns
IGHV1-46	ns	****	****	****	****	ns
IGHV4-61	***	ns	ns	****	***	ns
IGHV3-9	ns	ns	ns	ns	ns	ns
IGHV4-31	***	ns	ns	ns	*	ns
IGHV3-30	ns	ns	ns	ns	*	ns

*p<0.05; **p<0.01; ***p<0.001; ****p<0.0001

Supplementary Table 3. Frequency of clonotypes with convergent recombination in FL duplicate libraries

	FL56-1	FL56-2	FL66-1	FL66-2	FL76-1	FL76-2	FL86-1	FL86-2
unique clnt	4011	5243	1032	1275	4408	5626	3653	4753
unique convergent clnt	153	153	96	96	107	107	185	185
% unique convergent clnt	3.8	3	9.3	7.5	2.4	1.9	5	3.9

Supplementary Table 4. Repesentative examples of convergent VDJ recombination between duplicate FL libraries

* differences in VDJ annotation within (near-)identical nucleotide sequences are due to IMGT/V-QUEST's differential behaviour in accepted mutations depending on VH mutational status

junction	VH	DH	JH	FL-56-1	FL-56-2	junction	3'V-REGION	p	N1	D-REGION	N2	p	5'J-REGION	seq
CAKSNWGRRGFDYW	IGHV3-23*01F=IGHV3	IGHD7-27*01F	IGHJ4*02F	0.1744	0.2855	tgt gcg aaa agt aac tgg gga agg agg gga ttt gac tac tgg	tgtgcgaaa..		ag	.taactgggga	aggagggga		.....tttgactactgg	[illegible]
CAKSNWGRRGFDYW	IGHV3-21*04F	IGHD7-27*01F	IGHJ4*02F	0.0218	0.0124	tgt gcg aaa agt aac tgg gga agg agg gga ttt gac tac tgg	tgtgcga....		aaag	.taactgggga	aggagggga		.....tttgactactgg	[illegible]
CAKSNWGRRGFDYW	IGHV3-33*01F=IGHV3	IGHD7-27*01F	IGHJ4*02F	0.0073	0.0124	tgt gcg aaa agt aac tgg gga agg agg gga ttt gac tac tgg	tgtgcga....		aaag	.taactgggga	aggagggga		.....tttgactactgg	[illegible]
CARDLKLGWDAFDIW	IGHV5-51*01F	IGHD2-15*01F	IGHJ3*02F	0.0654	0.1738	tgt gcg aga gac ctg aaa ctg ggg tgg gat gct ttt gat atc tgg	tgtgcgaga..		gacct	..gaaactggggtgg...............			.gatgcttttgatatctgg	[illegible]
CARDLKLGWDAFDIW	IGHV5-51*01F	IGHD7-27*01F	IGHJ3*02F	0.0509	0.0248	tgt gcg aga gac ctg aaa ctg ggg tgg gat gct ttt gat atc tgg	tgtgcgaga..		gacctga	..aactgggg.	tgg		.gatgcttttgatatctgg	[illegible]
CAREVNANWNDAFDIW	IGHV6-1*01F=IGHV6-	IGHD2-21*01F	IGHJ3*02F	0.3051	0.3973	tgt gca agg gag gtg aat gcg aac tgg aat gat gct ttt gat atc tgg	tgtgcaag...			..........ggaggtgaatgc......	gaactgga	a	tgatgcttttgatatctgg	[illegible]
CAREVNANWNDAFDIW	IGHV6-1*01F=IGHV6-	IGHD1-1*01F	IGHJ3*02F	0.2906	0.2731	tgt gca agg gag gtg aat gcg aac tgg aat gat gct ttt gat atc tgg	tgtgcaaggga		ggtgaatgcg	.....aactggaa....			tgatgcttttgatatctgg	[illegible]
CARGYYYYMDVW	IGHV1-8*01F		IGHJ6*03F	0.7701	0.72	tgt gcg aga ggc tac tac tac tac atg gac gtc tgg	tgtgcgagagg						.......ctactactactacatggacgtctgg	[illegible]
CARGYYYYMDVW	IGHV1-2*02F		IGHJ6*03F	0.1961	0.1241	tgt gcg aga ggc tac tac tac tac atg gac gtc tgg	tgtgcgagag.		g				.......ctactactactacatggacgtctgg	[illegible]
CARGYYYYMDVW	IGHV1-8*02F		IGHJ6*03F	0.0654	0.0745	tgt gcg aga ggc tac tac tac tac atg gac gtc tgg	tgtgcgagagg						.......ctactactactacatggacgtctgg	[illegible]
CARGYYYYMDVW	IGHV1-69*13(F)		IGHJ6*03F	0.0726	0.0248	tgt gcg aga ggc tac tac tac tac atg gac gtc tgg	tgtgcgagag.		g				.......ctactactactacatggacgtctgg	[illegible]
CARGYYYYMDVW	IGHV1-24*01F		IGHJ6*03F	0.0073	0.0124	tgt gcg aga ggc tac tac tac tac atg gac gtc tgg	tgtgcgagag.		g				.......ctactactactacatggacgtctgg	[illegible]
CARLSVGYFDLW	IGHV5-51*01F	IGHD3-3*01F	IGHJ2*01F	0.1235	0.1986	tgt gcg aga ctt tcg gta ggg tac ttc gat ctc tgg	tgtgcgagac.			.........tttcggtag............			.....ggtacttcgatctctgg	[illegible]
CARLSVGYFDLW	IGHV5-51*01F	IGHD2-15*01F	IGHJ2*01F	0.1017	0.0248	tgt gcg aga ctt tcg gta ggg tac ttc gat ctc tgg	tgtgcgagac.		tttc	................ggtag..........			.....ggtacttcgatctctgg	[illegible]
CARLSVGYFDLW	IGHV5-10-1*01F=IGH	IGHD3-3*01F	IGHJ2*01F	0.0073	0.0124	tgt gcg aga ctt tcg gta ggg tac ttc gat ctc tgg	tgtgcgaga		c	.........tttcggtag............			.....ggtacttcgatctctgg	[illegible]
CARRLGSKKYFDLW	IGHV4-59*01F	IGHD7-27*01F	IGHJ2*01F	0.1162	0.211	tgt gcg agg aga ctg ggg agt aaa aag tac ttc gat ctc tgg	tgtgcgag...		gag	...actgggga	gtaaaaa		......gtacttcgatctctgg	[illegible]
CARRLGSKKYFDLW	IGHV4-59*01F	IGHD3-9*01F	IGHJ2*01F	0.1308	0.1614	tgt gcg agg aga ctg ggg agt aaa aag tac ttc gat ctc tgg	tgtgcgag...		ga	..............gactggggagtaaaa.	a		......gtacttcgatctctgg	[illegible]
CARRLGSKKYFDLW	IGHV4-39*07F	IGHD3-9*01F	IGHJ2*01F	0.0073	0.0372	tgt gcg agg aga ctg ggg agt aaa aag tac ttc gat ctc tgg	tgtgcgag...		ga	..............gactggggagtaaaa.	a		......gtacttcgatctctgg	[illegible]
CARRLGSKKYFDLW	IGHV4-34*03F	IGHD3-9*01F	IGHJ2*01F	0.0291	0.0124	tgt gcg agg aga ctg ggg agt aaa aag tac ttc gat ctc tgg	tgtgcgag...		ga	..............gactggggagtaaaa.	a		......gtacttcgatctctgg	[illegible]
CARRLGSKKYFDLW	IGHV4-61*08F	IGHD3-9*01F	IGHJ2*01F	0.0145	0.0124	tgt gcg agg aga ctg ggg agt aaa aag tac ttc gat ctc tgg	tgtgcgag...		ga	..............gactggggagtaaaa.	a		......gtacttcgatctctgg	[illegible]
CARRLGSKKYFDLW	IGHV4-31*09F	IGHD3-9*01F	IGHJ2*01F	0.0073	0.0124	tgt gcg agg aga ctg ggg agt aaa aag tac ttc gat ctt tgg	tgtgcgag...		ga	..............gactggggagtaaaa.	a		......gtacttcgatctttgg	[illegible]
				2.5647	2.8177									

junction	VH	DH	JH	FL-66-1	FL-66-2	junction	3'V-REGION	p	N1	D-REGION	N2	p	5'J-REGION	seq
CARDLGWFDPW	IGHV6-1*01F=IGHV6-	IGHD7-27*01F	IGHJ5*02F	7.8907	8.4185	tgt gca aga gat ctg ggg tgg ttc gac ccc tgg	tgtgcaagaga	t		....ctgggg.			.....tggttcgacccctgg	[illegible]
CARDLGWFDPW	IGHV6-1*01F=IGHV6-	IGHD3-10*01F	IGHJ5*02F	0.0561	0.0126	tgt gca agg gat ctg ggg tgg ttc gac ccc tgg	tgtgcaaggga	t		....ctgggg.			.....tggttcgacccctgg	[illegible]
CARDLGWFDPW	IGHV6-1*01F=IGHV6-	IGHD3-3*01F	IGHJ5*01F=I	0.024	0.0126	tgt gca aga gat ctg ggg tgg ttc gac ccc tgg	tgtgcaagaga	t		....ctgggg.			.....tggttcgacccctgg	[illegible]
CARGTGVDAFDIW	IGHV5-10-1*01F=IGH	IGHD7-27*01F	IGHJ3*02F	1.6823	1.5272	tgt gcg agg gga act ggg gtt gat gct ttt gat atc tgg	tgtgcgag.		ggg	..aactgggg.	t		tgatgcttttgatatctgg	[illegible]
CARGTGVDAFDIW	IGHV5-51*01F	IGHD7-27*01F	IGHJ3*02F	0.2964	0.2146	tgt gcg agg gga act ggg gtt gat gct ttt gat atc tgg	tgtgcgag.		ggg	..aactgggg.	t		tgatgcttttgatatctgg	[illegible]
CARGTGVDAFDIW	IGHV5-51*01F=IGHV5	IGHD7-27*01F	IGHJ3*02F	0.016	0.0126	tgt gcg agg gga act ggg gtt gat gct ttt gat atc tgg	tgtgcgag.		ggg	..aactgggg.	t		tgatgcttttgatatctgg	[illegible]
CARHGGIDAFDIW	IGHV5-51*01F	IGHD3-16*01F	IGHJ3*02F	5.2952	4.7331	tgt gcg aga cat ggg ggg att gat gct ttt gat atc tgg	tgtgcgagaca	tg		................ggggga.............	t		tgatgcttttgatatctgg	[illegible]
CARHGGIDAFDIW	IGHV5-10-1*01F=IGH	IGHD1-26*01F	IGHJ3*02F	0.1682	0.0757	tgt gcg aga cat ggg ggg att gat gct ttt gat atc tgg	tgtgcgaga		c	...atggggga........	t		tgatgcttttgatatctgg	[illegible]
CARHGGIDAFDIW	IGHV5-51*01F=IGHV5	IGHD3-16*01F	IGHJ3*02F	0.0641	0.0379	tgt gcg aga cat ggg ggg att gat gct ttt gat atc tgg	tgtgcgagaca	tg		................ggggga.............	t		tgatgcttttgatatctgg	[illegible]
CARHGGIDAFDIW	IGHV5-51*01F=IGHV5	IGHD3-16*01F	IGHJ3*02F	0.0401	0.0379	tgt gcg aga cat ggg ggg att gat gct ttt gat atc tgg	tgtgcgagaca	tg		................ggggga.............	t		tgatgcttttgatatctgg	[illegible]
CARHGGIDAFDIW	IGHV5-10-1*04F	IGHD1-26*01F	IGHJ3*02F	0.016	0.0126	tgt gcg aga cat ggg ggg att gat gct ttt gat atc tgg	tgtgcgaga		c	...atggggga........	t		tgatgcttttgatatctgg	[illegible]
CARHGGIDAFDIW	IGHV5-51*01F	IGHD2-21*01F	IGHJ3*02F	0.008	0.0126	tgt gcg aga cat ggg ggg att gac gct ttt gat atc tgg	tgtgcgagaca	tg		................ggggga.............	t		tgatgcttttgatatctgg	[illegible]
CARIHSSWDYYFDYW	IGHV2-70*01F	IGHD5-12*01F	IGHJ4*02F	6.1283	6.8661	tgt gca cgg ata cat tcg agc tgg gat tac tac ttt gac tac tgg	tgtgcacggatac		attcga	...........gctgggatt..			actactttgactactgg	[illegible]
CARIHSSWDYYFDYW	IGHV2-70*01F=IGHV2	IGHD5-12*01F	IGHJ4*02F	0.016	0.0379	tgt gca cgg ata cat tcg agc tgg gat tac tac ttt gac tac tgg	tgtgcacggatac		attcga	...........gctgggatt..			actactttgactactgg	[illegible]
CARIHSSWDYYFDYW	IGHV2-70*01F	IGHD6-13*01F	IGHJ4*02F	0.0721	0.0252	tgt gca cgg ata cat tcg agc tgg gac tac tac ttt gac tac tgg	tgtgcacggatac		attcg	...........agctgggac		t	actactttgactactgg	[illegible]
CARIHSSWDYYFDYW	IGHV2-70*01F	IGHD3-16*01F	IGHJ4*02F	0.008	0.0126	tgt gca cgg ata cat tcg agc tgg gat tat tac ttt gac tac tgg	tgtgcacggatac		attc	...................gagctgggattat...			..tactttgactactgg	[illegible]
CARIHSSWDYYFDYW	IGHV2-70*01F	IGHD5-12*01F	IGHJ4*01F=I	0.008	0.0126	tgt gca cgg ata cat tcg agc tgg gat tac tac ttt gac tac tgg	tgtgcacggatac		attcga	...........gctgggatt..			actactttgactactgg	[illegible]
CARLVILWYFDLW	IGHV5-51*01F	IGHD5-12*01F	IGHJ2*01F	2.3472	2.7136	tgt gcg aga ctg gtt ata ctg tgg tac ttc gat ctc tgg	tgtgcgagac.			.tggttatactg...........			....tggtacttcgatctctgg	[illegible]
CARLVILWYFDLW	IGHV5-10-1*01F=IGH	IGHD5-12*01F	IGHJ2*01F	0.0561	0.101	tgt gcg aga ctg gtt ata ctg tgg tac ttc gat ctc tgg	tgtgcgaga		c	.tggttatactg...........			....tggtacttcgatctctgg	[illegible]
CARLVILWYFDLW	IGHV5-51*03F	IGHD5-12*01F	IGHJ2*01F	0.0481	0.0126	tgt gcg aga ctg gtt ata ctg tgg tac ttc gat ctc tgg	tgtgcgaga		c	.tggttatactg...........			....tggtacttcgatctctgg	[illegible]
CARLVILWYFDLW	IGHV5-51*01F=IGHV5	IGHD5-12*01F	IGHJ2*01F	0.0401	0.0126	tgt gcg aga ctg gtt ata ctg tgg tac ttc gat ctc tgg	tgtgcgagac.			.tggttatactg...........			....tggtacttcgatctctgg	[illegible]
CARSYSSWYFDLW	IGHV2-70*01F	IGHD6-13*01F	IGHJ2*01F	8.3634	9.1001	tgt gca cgg tct tat agc agc tgg tac ttc gat ctc tgg	tgtgcacgg....		tct	...tatagcag..........			...ctggtacttcgatctctgg	[illegible]
CARSYSSWYFDLW	IGHV2-70*01F=IGHV2	IGHD6-13*01F	IGHJ2*01F	0.0401	0.0505	tgt gca cgg tct tat agc agc tgg tac ttc gat ctc tgg	tgtgcacgg....		tct	...tatagcag..........			...ctggtacttcgatctctgg	[illegible]
CARSYSSWYFDLW	IGHV2-70*01F	IGHD2-2*01F	IGHJ2*01F	0.024	0.0252	tgt gca cgg tct tat agt agc tgg tac ttc gat ctc tgg	tgtgcacgg....		tc	......ttatagtag...............			...ctggtacttcgatctctgg	[illegible]
CARSYSSWYFDLW	IGHV2-70*11F	IGHD6-13*01F	IGHJ2*01F	0.0481	0.0126	tgt gca cgg tct tat agc agc tgg tac ttc gat ctc tgg	tgtgcacgg....		tct	...tatagcag..........			...ctggtacttcgatctctgg	[illegible]
				23.1035	24.1196									

junction	VH	DH	JH	FL-76-1	FL-76-2	junction	3'V-REGION	p	N1	D-REGION	N2	p	5'J-REGION	seq
CAHRTGDMYDYW	IGHV2-5*02F	IGHD7-27*01F	IGHJ4*02F	0.7043	0.7635	tgt gca cac aga act ggg gat atg tat gac tac tgg	tgtgcacacaga.			...actgggga	t	atgta	.......tgactactgg	[illegible]
CAHRTGDMYDYW	IGHV2-70*11F	IGHD7-27*01F	IGHJ4*02F	0.0749	0.1355	tgt gca cac aga act ggg gat atg tat gac tac tgg	tgtgcac......		acag	..aactgggga	t	atgta	.......tgactactgg	[illegible]
CAHRTGDMYDYW	IGHV2-70*01F	IGHD7-27*01F	IGHJ4*02F	0.0599	0.0739	tgt gca cac aga act ggg gat atg tat gac tac tgg	tgtgcac......		acag	..aactgggga	t	atgta	.......tgactactgg	[illegible]
CAHRTGDMYDYW	IGHV2-5*08F	IGHD7-27*01F	IGHJ4*02F	0.0524	0.0616	tgt gca cac aga act ggg gat atg tat gac tac tgg	tgtgcacacaga.			...actgggga	t	atgta	.......tgactactgg	[illegible]
CAHRTGDMYDYW	IGHV2-70*12F	IGHD7-27*01F	IGHJ4*02F	0.0075	0.0369	tgt gca cac aga act ggg gat atg tat gac tac tgg	tgtgcacacaga.			...actgggga	t	atgta	.......tgactactgg	[illegible]
CAHRTGDMYDYW	IGHV2-70*11F=IGHV2	IGHD7-27*01F	IGHJ4*02F	0.015	0.0123	tgt gca cac aga act ggg gat atg tat gac tac tgg	tgtgcac......		acag	..aactgggga	t	atgta	.......tgactactgg	[illegible]
CAREWNAFDIW	IGHV6-1*01F=IGHV6-	IGHD3-3*01F	IGHJ3*02F	1.0564	1.2562	tgt gca agg gaa tgg aat ... gct ttt gat atc tgg	tgtgcaaggga		a	........tgga.....			..atgcttttgatatctgg	[illegible]
CAREWNAFDIW	IGHV6-1*01F=IGHV6-	IGHD1-1*01F	IGHJ3*02F	0.959	0.9483	tgt gca agg gaa tgg aat ... gct ttt gat atc tgg	tgtgcaaggga		a	........tgga.....			..atgcttttgatatctgg	[illegible]
CARGGRRPDYW	IGHV1-8*01F	IGHD2-15*01F	IGHJ4*02F	0.2472	0.234	tgt gcg agg ggg ggt agg cga ccc gac tac tgg	tgtgcgagggg			..........gggtaggcga...........		ccc	........gactactgg	[illegible]
CARGGRRPDYW	IGHV1-8*01F	IGHD3-10*01F	IGHJ4*02F	0.2323	0.1601	tgt gcg agg ggg ggt agg cga ccc gac tac tgg	tgtgcgagggg			..........gggtaggcga...........		ccc	........gactactgg	[illegible]
CARGGRRPDYW	IGHV1-18*01F	IGHD3-16*01F	IGHJ4*02F	0.0524	0.0369	tgt gcg agg ggg ggt agg cga ccc gac tac tgg	tgtgcgag...			..................gggggtaggc.......	gaccc		........gactactgg	[illegible]
CARGGRRPDYW	IGHV1-8*02F	IGHD3-10*01F	IGHJ4*02F	0.0899	0.0369	tgt gcg agg ggg ggt agg cga ccc gac tac tgg	tgtgcgagggg			..........gggtaggcga...........	ccc		........gactactgg	[illegible]
CARGGRRPDYW	IGHV1-18*01F	IGHD2-15*01F	IGHJ4*02F	0.0075	0.0123	tgt gcg agg ggg ggt agg cga ccc gac tac tgg	tgtgcgag...			..................gggggtaggc.......	gaccc		........gactactgg	[illegible]
CARGGRRPDYW	IGHV1-18*04F	IGHD3-16*01F	IGHJ4*02F	0.0075	0.0123	tgt gcg agg ggg ggt agg cga ccc gac tac tgg	tgtgcgag...			..................gggggtaggc.......	gaccc		........gactactgg	[illegible]
CARGGRRPDYW	IGHV1-2*04F	IGHD3-16*01F	IGHJ4*02F	0.0075	0.0123	tgt gcg agg ggg ggt agg cga ccc gac tac tgg	tgtgcgag...			..................gggggtaggc.......	gaccc		........gactactgg	[illegible]
CARGGRRPDYW	IGHV1-3*01F	IGHD3-16*01F	IGHJ4*02F	0.0075	0.0123	tgt gcg agg gga ggt agg cga ccc gac tac tgg	tgtgcgag...			..................gggaggtaggc.......	gaccc		........gactactgg	[illegible]
CARGGRRPDYW	IGHV1-69*04F=IGHV1	IGHD3-16*01F	IGHJ4*02F	0.0225	0.0123	tgt gcg agg ggg ggt agg cga ccc gac tac tgg	tgtgcgag...			..................gggggtaggc.......	gaccc		........gactactgg	[illegible]
CARGGRRPDYW	IGHV1-24*01F	IGHD2-15*01F	IGHJ4*02F	0.03	0.0123	tgt gcg agg ggg ggt agg cga ccc gac tac tgg	tgtgc......		g	..........aggggggtag..........	ccc		........gactactgg	[illegible]
CARHASWGYIPYAFDIW	IGHV4-39*01F	IGHD3-16*02F	IGHJ3*02F	0.2248	0.2463	tgt gcg aga cat gca agc tgg gga tat atc ccc tat gct ttt gat atc tgg	tgtgcgagaca	tg	caagct	.................ggggatatatc........	ccct		..atgcttttgatatctgg	[illegible]
CARHASWGYIPYAFDIW	IGHV4-34*01F=IGHV4	IGHD3-16*02F	IGHJ3*02F	0.0225	0.0369	tgt gcg aga cat gca agc tgg gga tat atc ccc tat gct ttt gat atc tgg	tgtgcgaga..		catgcaagct	.................ggggatatatc........	ccct		..atgcttttgatatctgg	[illegible]
CARHASWGYIPYAFDIW	IGHV4-59*08F	IGHD3-16*02F	IGHJ3*02F	0.045	0.0369	tgt gcg aga cat gca agc tgg gga tat atc ccc tat gct ttt gat atc tgg	tgtgcgagaca	tg	caagct	.................ggggatatatc........	ccct		..atgcttttgatatctgg	[illegible]
CARHSHKITGDNAFDIW	IGHV4-59*08F	IGHD7-27*01F	IGHJ3*02F	0.2997	0.2094	tgt gcg aga cat tct cat aaa ata act ggg gat aat gct ttt gat atc tgg	tgtgcgagaca	t	tctcataaaa	.taactgggga	t	a	..atgcttttgatatctgg	[illegible]
CARHSHKITGDNAFDIW	IGHV4-39*03F	IGHD7-27*01F	IGHJ3*02F	0.0075	0.0369	tgt gcg aga cat tct cat aaa ata act ggg gat aat gct ttt gat atc tgg	tgtgcgagaca	t	tctcataaaa	.taactgggga	t	a	..atgcttttgatatctgg	[illegible]
CARHSHKITGDNAFDIW	IGHV4-59*01F	IGHD7-27*01F	IGHJ3*02F	0.0225	0.0369	tgt gcg aga cat tct cat aaa ata act ggg gat aat gct ttt gat atc tgg	tgtgcgaga..		cattctcataaaa	.taactgggga	t	a	..atgcttttgatatctgg	[illegible]
CARHSHKITGDNAFDIW	IGHV4-34*03F	IGHD7-27*01F	IGHJ3*02F	0.015	0.0246	tgt gcg aga cat tct cat aaa ata act ggg gat aat gct ttt gat atc tgg	tgtgcgaga..		cattctcataaaa	.taactgggga	t	a	..atgcttttgatatctgg	[illegible]
CARHSHKITGDNAFDIW	IGHV4-39*03F=IGHV4	IGHD7-27*01F	IGHJ3*02F	0.0225	0.0246	tgt gcg aga cat tct cat aaa ata act ggg gat aat gct ttt gat atc tgg	tgtgcgagaca	t	tctcataaaa	.taactgggga	t	a	..atgcttttgatatctgg	[illegible]
CARHSHKITGDNAFDIW	IGHV4-61*05F	IGHD7-27*01F	IGHJ3*02F	0.0225	0.0123	tgt gcg aga cat tct cat aaa ata act ggg gat aat gct ttt gat atc tgg	tgtgcgaga		cattctcataaaa	.taactgggga	t	a	..atgcttttgatatctgg	[illegible]
CARVNWGFDYW	IGHV2-70*11F	IGHD7-27*01F	IGHJ4*02F	1.0939	0.9483	tgt gca cgg gtt aac tgg gga ttt gac tac tgg	tgtgcacgg....		gt	.taactgggga			.....tttgactactgg	[illegible]
CARVNWGFDYW	IGHV2-70*01F	IGHD7-27*01F	IGHJ4*02F	0.1948	0.2463	tgt gca cgg gtt aac tgg gga ttt gac tac tgg	tgtgcacgg....		gt	.taactgggga			.....tttgactactgg	[illegible]
CARVNWGFDYW	IGHV2-5*02F	IGHD7-27*01F	IGHJ4*02F	0.3521	0.1232	tgt gca cgg gtt aac tgg gga ttt gac tac tgg	tgtgcac......		gggt	.taactgggga			.....tttgactactgg	[illegible]
CARVNWGFDYW	IGHV2-5*08F	IGHD7-27*01F	IGHJ4*02F	0.03	0.0123	tgt gca cgg gtt aac tgg gga ttt gac tac tgg	tgtgcac......		gggt	.taactgggga			.....tttgactactgg	[illegible]
				2.5776	2.1425									

junction	VH	DH	JH	FL-86-1	FL-86-2	junction	3'V-REGION	p	N1	D-REGION	N2	p	5'J-REGION	seq
CAKAGGIAAAGNWFDPW	IGHV3-23*01F=IGHV3	IGHD6-13*01F	IGHJ5*02F	0.3703	0.2195	tgt gcg aaa gct ggg ggt ata gca gca gct ggt aac tgg ttc gac ccc tgg	tgtgcgaaag.		ctgg	gggtatagcagcagctggt..			..aactggttcgacccctgg	[illegible]
CAKAGGIAAAGNWFDPW	IGHV3-33*01F=IGHV3	IGHD6-13*01F	IGHJ5*02F	0.0135	0.0116	tgt gcg aaa gct ggg ggt ata gca gca gct ggt aac tgg ttc gac ccc tgg	tgtgcgaaag.		ctgg	gggtatagcagcagctggt..			..aactggttcgacccctgg	[illegible]
CAKAGGIAAAGNWFDPW	IGHV3-30*03F=IGHV3	IGHD6-13*01F	IGHJ5*02F	0.0067	0.0116	tgt gcg aaa gct ggg ggt ata gca gca gct ggt aac tgg ttc gac ccc tgg	tgtgcgaaag.		ctgg	gggtatagcagcagctggt..			..aactggttcgacccctgg	[illegible]
CARDYYYYGMDVW	IGHV4-59*01F		IGHJ6*02F	0.3905	0.3697	tgt gcg aga gat tac tac tac tac ggt atg gac gtc tgg	tgtgcgagaga	t					.....tactactactacggtatggacgtctgg	[illegible]
CARDYYYYGMDVW	IGHV4-39*03F=IGHV4-39*07F		IGHJ6*02F	0.0673	0.0347	tgt gcg aga gat tac tac tac tac ggt atg gac gtc tgg	tgtgcgaga..		gat				.....tactactactacggtatggacgtctgg	[illegible]
CARDYYYYGMDVW	IGHV4-61*01F		IGHJ6*02F	0.0404	0.0116	tgt gcg aga gat tac tac tac tac ggt atg gac gtc tgg	tgtgcgagaga	t					.....tactactactacggtatggacgtctgg	[illegible]
CARDYYYYGMDVW	IGHV4-61*02F		IGHJ6*02F	0.0404	0.0116	tgt gcg aga gat tac tac tac tac ggt atg gac gtc tgg	tgtgcgagaga	t					.....tactactactacggtatggacgtctgg	[illegible]
CARDYYYYGMDVW	IGHV4-39*07F		IGHJ6*02F	0.0337	0.0347	tgt gcg aga gat tac tac tac tac ggt atg gac gtc tgg	tgtgcgagaga	t					.....tactactactacggtatggacgtctgg	[illegible]
CARDYYYYGMDVW	IGHV4-31*03F=IGHV4-31*04F		IGHJ6*02F	0.0067	0.0116	tgt gcg aga gat tac tac tac tac ggt atg gac gtc tgg	tgtgcgagaga	t					.....tactactactacggtatggacgtctgg	[illegible]
CARDYYYYGMDVW	IGHV4-39*01F		IGHJ6*02F	0.0067	0.0116	tgt gcg aga gat tac tac tac tac ggt atg gac gtc tgg	tgtgcgaga..		gat				.....tactactactacggtatggacgtctgg	[illegible]
CARDYYYYGMDVW	IGHV4-59*01F=IGHV4-59*02F		IGHJ6*02F	0.0067	0.0116	tgt gcg aga gat tac tac tac tac ggt atg gac gtc tgg	tgtgcgagaga	t					.....tactactactacggtatggacgtctgg	[illegible]
CARGKLTPDYW	IGHV4-59*01F	IGHD1-26*01F	IGHJ4*02F	0.4713	0.4159	tgt gcg aga ggg aag cta aca cct gac tac tgg	tgtgcgagag.			........ggaagcta....	acacc		.......tgactactgg	[illegible]
CARGKLTPDYW	IGHV4-39*07F	IGHD1-26*01F	IGHJ4*02F	0.0471	0.0116	tgt gcg aga ggg aag cta aca cct gac tac tgg	tgtgcgagag.			........ggaagcta....	acacc		.......tgactactgg	[illegible]
CARGKLTPDYW	IGHV4-61*01F	IGHD1-26*01F	IGHJ4*02F	0.0269	0.0578	tgt gcg aga ggg aag cta aca cct gac tac tgg	tgtgcgagag.			........ggaagcta....	acacc		.......tgactactgg	[illegible]
CARGKLTPDYW	IGHV4-4*07F	IGHD1-26*01F	IGHJ4*02F	0.0269	0.0231	tgt gcg aga ggg aag cta aca cct gac tac tgg	tgtgcgagag.			........ggaagcta....	acacc		.......tgactactgg	[illegible]
CARGKLTPDYW	IGHV4-34*03F	IGHD3-16*01F	IGHJ4*02F	0.0269	0.0116	tgt gcg aga ggg aag cta aca cct gac tac tgg	tgtgcgagagg		gaa	...........................gctaacacc			.......tgactactgg	[illegible]
CARGKLTPDYW	IGHV4-61*02F	IGHD1-26*01F	IGHJ4*02F	0.0269	0.0116	tgt gcg aga ggg aag cta aca cct gac tac tgg	tgtgcgagag.			........ggaagcta....	acacc		.......tgactactgg	[illegible]
CARGKLTPDYW	IGHV4-39*01F	IGHD1-26*01F	IGHJ4*02F	0.0135	0.0231	tgt gcg aga ggg aag cta aca cct gac tac tgg	tgtgcgaga..		g	........ggaagcta....	acacc		.......tgactactgg	[illegible]
CARGKLTPDYW	IGHV4-39*03F=IGHV4	IGHD1-26*01F	IGHJ4*02F	0.0135	0.0231	tgt gcg aga ggg aag cta aca cct gac tac tgg	tgtgcgaga..		g	........ggaagcta....	acacc		.......tgactactgg	[illegible]
CARGKLTPDYW	IGHV4-59*01F=IGHV4	IGHD1-26*01F	IGHJ4*02F	0.0135	0.0116	tgt gcg aga ggg aag cta aca cct gac tac tgg	tgtgcgagag.			........ggaagcta....	acacc		.......tgactactgg	[illegible]
CARGKLTPDYW	IGHV4-61*05F	IGHD1-26*01F	IGHJ4*02F	0.0067	0.0231	tgt gcg aga ggg aag cta aca cct gac tac tgg	tgtgcgaga		g	........ggaagcta....	acacc		.......tgactactgg	[illegible]
CARKLGGAFDIW	IGHV6-1*01F=IGHV6-	IGHD7-27*01F	IGHJ3*02F	1.9793	3.8355	tgt gca aga aaa ctg ggg ggt gct ttt gat atc tgg	tgtgcaaga..		a	..aactgggg.	gg		...tgcttttgatatctgg	[illegible]
CARKLGGAFDIW	IGHV6-1*01F=IGHV6-	IGHD3-16*01F	IGHJ3*02F	0.0067	0.0116	tgt gca aga aag ctg ggg ggt gct ttt gat atc tgg	tgtgcaaga..		aagc	...............tggggg...............	g		...tgcttttgatatctgg	[illegible]
CARRRYWYFDLW	IGHV4-39*01F	IGHD6-19*01F	IGHJ2*01F	0.5049	0.4043	tgt gcg aga cgc cgt tac tgg tac ttc gat ctc tgg	tgtgcgagac.			.......gccgt.........			.tactggtacttcgatctctgg	[illegible]
CARRRYWYFDLW	IGHV4-59*08F	IGHD6-19*01F	IGHJ2*01F	0.0741	0.1386	tgt gcg aga cgc cgt tac tgg tac ttc gat ctc tgg	tgtgcgagac.			.......gccgt.........			.tactggtacttcgatctctgg	[illegible]
CARRRYWYFDLW	IGHV4-39*07F	IGHD6-19*01F	IGHJ2*01F	0.0269	0.0116	tgt gcg aga cgc cgt tac tgg tac ttc gat ctc tgg	tgtgcgaga..		c	.......gccgt.........			.tactggtacttcgatctctgg	[illegible]
CARRRYWYFDLW	IGHV4-34*01F=IGHV4	IGHD6-19*01F	IGHJ2*01F	0.0202	0.0116	tgt gcg aga cgc cgt tac tgg tac ttc gat ctc tgg	tgtgcgaga..		c	.......gccgt.........			.tactggtacttcgatctctgg	[illegible]
CARRRYWYFDLW	IGHV4-4*07F	IGHD6-19*01F	IGHJ2*01F	0.0067	0.0231	tgt gcg aga cgc cgt tac tgg tac ttc gat ctc tgg	tgtgcgaga..		c	.......gccgt.........			.tactggtacttcgatctctgg	[illegible]
CARRRYWYFDLW	IGHV4-31*08F	IGHD6-19*01F	IGHJ2*01F	0.0067	0.0116	tgt gcg aga cgc cgt tac tgg tac ttc gat ctc tgg	tgtgcgaga..		c	.......gccgt.........			.tactggtacttcgatctctgg	[illegible]
CARRRYWYFDLW	IGHV4-59*01F	IGHD6-19*01F	IGHJ2*01F	0.0067	0.0116	tgt gcg aga cgc cgt tac tgg tac ttc gat ctc tgg	tgtgcgaga..		c	.......gccgt.........			.tactggtacttcgatctctgg	[illegible]
CARRRYWYFDLW	IGHV4-59*04F	IGHD6-19*01F	IGHJ2*01F	0.0067	0.0116	tgt gcg aga cgc cgt tac tgg tac ttc gat ctc tgg	tgtgcgaga..		c	.......gccgt.........			.tactggtacttcgatctctgg	[illegible]
CARRRYWYFDLW	IGHV4-61*01F	IGHD6-19*01F	IGHJ2*01F	0.0067	0.0116	tgt gcg aga cgc cgt tac tgg tac ttc gat ctc tgg	tgtgcgaga..		c	.......gccgt.........			.tactggtacttcgatctctgg	[illegible]
CTINWGSYGAFDIW	IGHV1-18*01F	IGHD7-27*01F	IGHJ3*02F	1.1243	0.9242	tgt act att aac tgg gga tcc tac ggg gct ttt gat atc tgg	tgt........		actat	.taactgggga	tacggg	tcc	....gcttttgatatctgg	[illegible]
CTINWGSYGAFDIW	IGHV1-8*01F	IGHD7-27*01F	IGHJ3*02F	0.0875	0.104	tgt act att aac tgg gga tcc tac ggg gct ttt gat atc tgg	tgt........		actat	.taactgggga	tacggg	tcc	....gcttttgatatctgg	[illegible]
CTINWGSYGAFDIW	IGHV1-2*02F	IGHD7-27*01F	IGHJ3*02F	0.0741	0.0578	tgt act att aac tgg gga tcc tac ggg gct ttt gat atc tgg	tgt........		actat	.taactgggga	tacggg	tcc	....gcttttgatatctgg	[illegible]
CTINWGSYGAFDIW	IGHV1-18*04F	IGHD7-27*01F	IGHJ3*02F	0.0741	0.0231	tgt act att aac tgg gga tcc tac ggg gct ttt gat atc tgg	tgt........		actat	.taactgggga	tacggg	tcc	....gcttttgatatctgg	[illegible]
CTINWGSYGAFDIW	IGHV1-69*13(F)	IGHD7-27*01F	IGHJ3*02F	0.0404	0.0231	tgt act att aac tgg gga tcc tac ggg gct ttt gat atc tgg	tgt........		actat	.taactgggga	tacggg	tcc	....gcttttgatatctgg	[illegible]
CTINWGSYGAFDIW	IGHV1-8*02F	IGHD7-27*01F	IGHJ3*02F	0.0202	0.0347	tgt act att aac tgg gga tcc tac ggg gct ttt gat atc tgg	tgt........		actat	.taactgggga	tacggg	tcc	....gcttttgatatctgg	[illegible]
CTINWGSYGAFDIW	IGHV1-2*02F=IGHV1-	IGHD7-27*01F	IGHJ3*02F	0.0135	0.0116	tgt act att aac tgg gga tcc tac ggg gct ttt gat atc tgg	tgt........		actat	.taactgggga	tacggg	tcc	....gcttttgatatctgg	[illegible]
CTINWGSYGAFDIW	IGHV1-69*04F=IGHV1	IGHD7-27*01F	IGHJ3*02F	0.0067	0.0116	tgt act att aac tgg gga tcc tac ggg gct ttt gat atc tgg	tgt........		actat	.taactgggga	tacggg	tcc	....gcttttgatatctgg	[illegible]
				4.1066	5.7075									

Supplementary Table 5. Repesentative examples of convergent recombination between duplicate FL libraries

Arbitrarily selected examples of pairs of clonotypes showing evidence of convergent evolution from Suppl Table 4 (coloured the same) were analysed with IMGT/V-QUEST.

The full VH alignments of the two assigned VH genes and alleles are shown for either clonotype (#1 and #2).

FL-56-1 FL-56-2

		junction	VH	DH	JH	FL-56-1	FL-56-2	junction
#1	FL-56-1	CARGYYYYMDVW	IGHV1-8*01F		IGHJ6*03F	0.7701	0.72	tgt gcg aga ggc tac tac tac tac atg gac gtc tgg
#2	FL-56-2	CARGYYYYMDVW	IGHV1-2*02F		IGHJ6*03F	0.1961	0.1241	tgt gcg aga ggc tac tac tac tac atg gac gtc tgg

IMGT/V-QUEST

```
#1             [illegible]
M99637 Homsap IGHV1-8*01 F     [illegible]
X62106 Homsap IGHV1-2*02 F     [illegible]
               <---- FR1-IMGT ---->  CDR1-IMGT  <---- FR2-IMGT ---->  CDR2-IMGT  <---- FR3-IMGT ---->  CDR3-IMGT
#2             [illegible]
X62106 Homsap IGHV1-2*02 F     [illegible]
M99637 Homsap IGHV1-8*01 F     [illegible]
```

		junction	VH	DH	JH	FL-56-1	FL-56-2	junction
#1	FL-56-1	CARRLGSKKYFDLW	IGHV4-59*01F	IGHD7-27*01F	IGHJ2*01F	0.1162	0.211	tgt gcg agg aga ctg ggg agt aaa aag tac ttc gat ctc tgg
#2	FL-56-2	CARRLGSKKYFDLW	IGHV4-39*07F	IGHD3-9*01F	IGHJ2*01F	0.0073	0.0372	tgt gcg agg aga ctg ggg agt aaa aag tac ttc gat ctc tgg

IMGT/V-QUEST

```
#1             [illegible]
AB019438 Homsap IGHV4-59*01 F  [illegible]
AM940222 Homsap IGHV4-39*07 F  [illegible]
               <---- FR1-IMGT ---->  CDR1-IMGT  <---- FR2-IMGT ---->  CDR2-IMGT  <---- FR3-IMGT ---->  CDR3-IMGT
#2             [illegible]
AM940222 Homsap IGHV4-39*07 F  [illegible]
AB019438 Homsap IGHV4-59*01 F  [illegible]
```

FL-66-1 FL-66-2

		junction	VH	DH	JH	FL-56-1	FL-56-2	junction
#1	FL-66-1	CARGTGVDAFDIW	IGHV5-10-1*01F=I	IGHD7-27*01F	IGHJ3*02F	1.6823	1.5272	tgt gcg agg gga act ggg gtt gat gct ttt gat atc tgg
#2	FL-66-2	CARGTGVDAFDIW	IGHV5-51*01F		IGHJ3*02F	0.2964	0.2146	tgt gcg agg gga act ggg gtt gat gct ttt gat atc tgg

IMGT/V-QUEST

```
#1             [illegible]
X92227 Homsap IGHV5-10-1*01 F  [illegible]
M99686 Homsap IGHV5-51*01 F    [illegible]
               <---- FR1-IMGT ---->  CDR1-IMGT  <---- FR2-IMGT ---->  CDR2-IMGT  <---- FR3-IMGT ---->  CDR3-IMGT
#2             [illegible]
M99686 Homsap IGHV5-51*01 F    [illegible]
X92227 Homsap IGHV5-10-1*01 F  [illegible]
```

		junction	VH	DH	JH	FL-56-1	FL-56-2	junction
#1	FL-66-1	CARLVILWYFDLW	IGHV5-51*01F		IGHJ2*01F	2.3472	2.7136	tgt gcg aga ctg gtt ata ctg tgg tac ttc gat ctc tgg
#2	FL-66-2	CARLVILWYFDLW	IGHV5-10-1*01F=I	IGHD5-12*01F	IGHJ2*01F	0.0561	0.101	tgt gcg aga ctg gtt ata ctg tgg tac ttc gat ctc tgg

IMGT/V-QUEST

```
#1             [illegible]
M99686 Homsap IGHV5-51*01 F    [illegible]
X92227 Homsap IGHV5-10-1*01 F  [illegible]
               <---- FR1-IMGT ---->  CDR1-IMGT  <---- FR2-IMGT ---->  CDR2-IMGT  <---- FR3-IMGT ---->  CDR3-IMGT
#2             [illegible]
X92227 Homsap IGHV5-10-1*01 F  [illegible]
M99686 Homsap IGHV5-51*01 F    [illegible]
```

FL-76-1 FL-76-2

		junction	VH	DH	JH	FL-56-1	FL-56-2	junction
#1	FL-76-1	CAHRTGDMYDYW	IGHV2-5*02F	IGHD7-27*01F	IGHJ4*02F	0.7043	0.7635	tgt gca cac aga act ggg gat atg tat gac tac tgg
#2	FL-76-2	CAHRTGDMYDYW	IGHV2-70*11F	IGHD7-27*01F	IGHJ4*02F	0.0749	0.1355	tgt gca cac aga act ggg gat atg tat gac tac tgg

IMGT/V-QUEST

```
1              [illegible]
KF698731 Homsap IGHV2-5*02 F   [illegible]
L21967 Homsap IGHV2-70*11 F    [illegible]
               <---- FR1-IMGT ---->  CDR1-IMGT  <---- FR2-IMGT ---->  CDR2-IMGT  <---- FR3-IMGT ---->  CDR3-IMGT
2              [illegible]
L21967 Homsap IGHV2-70*11 F    [illegible]
KF698731 Homsap IGHV2-5*02 F   [illegible]
```

		junction	VH	DH	JH	FL-56-1	FL-56-2	junction
#1	FL-76-1	CARVNWGFDYW	IGHV2-70*11F	IGHD7-27*01F	IGHJ4*02F	1.0939	0.9483	tgt gca cgg gtt aac tgg gga ttt gac tac tgg
#2	FL-76-2	CARVNWGFDYW	IGHV2-5*02F	IGHD7-27*01F	IGHJ4*02F	0.3521	0.1232	tgt gca cgg gtt aac tgg gga ttt gac tac tgg

IMGT/V-QUEST

```
#1             [illegible]
L21967 Homsap IGHV2-70*11 F    [illegible]
KF698731 Homsap IGHV2-5*02 F   [illegible]
               <---- FR1-IMGT ---->  CDR1-IMGT  <---- FR2-IMGT ---->  CDR2-IMGT  <---- FR3-IMGT ---->  CDR3-IMGT
#2             [illegible]
KF698731 Homsap IGHV2-5*02 F   [illegible]
L21967 Homsap IGHV2-70*11 F    [illegible]
```

FL-86-1 FL-86-2

		junction	VH	DH	JH	FL-56-1	FL-56-2	junction
#1	FL-86-1	CARGKLTPDYW	IGHV4-59*01F	IGHD1-26*01F	IGHJ4*02F	0.4713	0.4159	tgt gcg aga ggg aag cta aca cct gac tac tgg
#2	FL-86-2	CARGKLTPDYW	IGHV4-39*07F	IGHD1-26*01F	IGHJ4*02F	0.0471	0.0116	tgt gcg aga ggg aag cta aca cct gac tac tgg

IMGT/V-QUEST

```
#1             [illegible]
AB019438 Homsap IGHV4-59*01 F  [illegible]
AM940222 Homsap IGHV4-39*07 F  [illegible]
               <---- FR1-IMGT ---->  CDR1-IMGT  <---- FR2-IMGT ---->  CDR2-IMGT  <---- FR3-IMGT ---->  CDR3-IMGT
#2             [illegible]
AM940222 Homsap IGHV4-39*07 F  [illegible]
AB019438 Homsap IGHV4-59*01 F  [illegible]
```

		junction	VH	DH	JH	FL-56-1	FL-56-2	junction
#1	FL-86-1	CTINWGSYGAFDIW	IGHV1-18*01F	IGHD7-27*01F	IGHJ3*02F	1.1243	0.9242	tgt act att aac tgg gga tcc tac ggg gct ttt gat atc tgg
#2	FL-86-2	CTINWGSYGAFDIW	IGHV1-8*01F	IGHD7-27*01F	IGHJ3*02F	0.0875	0.104	tgt act att aac tgg gga tcc tac ggg gct ttt gat atc tgg

IMGT/V-QUEST

```
#1             [illegible]
M99641 Homsap IGHV1-18*01 F    [illegible]
M99637 Homsap IGHV1-8*01 F     [illegible]
               <---- FR1-IMGT ---->  CDR1-IMGT  <---- FR2-IMGT ---->  CDR2-IMGT  <---- FR3-IMGT ---->  CDR3-IMGT
#2             [illegible]
M99637 Homsap IGHV1-8*01 F     [illegible]
M99641 Homsap IGHV1-18*01 F    [illegible]
```

Supplementary Table 6. Immunogenetic features of shared clonotypes between FL and FBM

junction	VH	DH	JH	CDR3L	#	FL, %	FBM, %
CARISGDAFDIW	IGHV2-70*01F	IGHD3-10*01F	IGHJ3*02F	12	1	0.0674	0.0223
CARGYCSGGSCYYFDYW	IGHV6-1*01F=IGHV6-1*02F	IGHD2-15*01F	IGHJ4*02F	17	2	0.0524	0.0111
CARVTGDAFDIW	IGHV6-1*01F=IGHV6-1*02F	IGHD7-27*01F	IGHJ3*02F	12	3	0.0504	0.0334
CARLTGDAFDIW	IGHV6-1*01F=IGHV6-1*02F	IGHD7-27*01F	IGHJ3*02F	12	4	0.0399	0.0228
CARDWYFDLW	IGHV3-33*01F=IGHV3-33*06F		IGHJ2*01F	10	5	0.0372	0.0108
CARDNWGSAFDIW	IGHV6-1*01F=IGHV6-1*02F	IGHD7-27*01F	IGHJ3*02F	13	6	0.03	0.0111
CARDGFDYW	IGHV3-7*01F	IGHD1-26*01F	IGHJ4*02F	9	7	0.016	0.0108
CAKGYSSSWYFDYW	IGHV3-30*03F=IGHV3-30*18F=IGHV3-30-5*01F	IGHD6-13*01F	IGHJ4*02F	14	8	0.015	0.0108
CARGFDYW	IGHV1-69*13(F)	IGHD1-26*01F	IGHJ4*02F	8	9	0.0124	0.0108
CARGGYSGYDDAFDIW	IGHV6-1*01F=IGHV6-1*02F	IGHD5-12*01F	IGHJ3*02F	16	10	0.0123	0.0111
CAREQTGDAFDIW	IGHV1-2*04F	IGHD7-27*01F	IGHJ3*02F	13	11	0.0099	0.0108
CAREANSDAFDIW	IGHV1-8*01F	IGHD4-23*01ORF	IGHJ3*02F	13	12	0.0099	0.0111
CARGRTGDAFDIW	IGHV6-1*01F=IGHV6-1*02F	IGHD1-1*01F	IGHJ3*02F	13	13	0.0075	0.0108
CARDAFDIW	IGHV1-2*04F		IGHJ3*02F	9	14	0.0075	0.0108
CARDGFDYW	IGHV3-33*01F=IGHV3-33*06F	IGHD1-26*01F	IGHJ4*02F	9	15	0.008	0.0217
**% of total repertoire**						**0.3758**	**0.2202**

Supplementary Table 7. Immunogenetic features of shared clonotypes between FL and postnatal samples

junction	VH	DH	JH	CDR3L	#	FL, %	cPB, %	aPB, %
**CARAKRGYYGMDVW**	IGHV5-51*01F=IGHV5-51*02F	IGHD5-24*01ORF	IGHJ6*02F	14	1	5.6772	0	0.0257
**CARNSGGTFDYW**	IGHV6-1*01F=IGHV6-1*02F	IGHD2-15*01F	IGHJ4*02F	12	2	2.7727	0	0.036
**CVHRLSTLRGDIPYFDYW**	IGHV2-5*02F	IGHD3-16*02F	IGHJ4*02F	18	3	2.503	0	0.2728
**CARHSAVGGTRHGMDVW**	IGHV5-51*01F	IGHD6-19*01F	IGHJ6*02F	17	4	1.5306	0	0.0154
**CARGRHCSSTSCYFPLFDYW**	IGHV4-59*01F	IGHD2-2*01F	IGHJ4*02F	20	5	1.1856	0	0.0051
**CARAHGSGWETGAFDIW**	IGHV6-1*01F=IGHV6-1*02F	IGHD6-19*01F	IGHJ3*02F	17	6	0.6461	0	0.0051
CARDGGYDWGTPRHFFDYW	IGHV1-18*01F	IGHD5-12*01F	IGHJ4*02F	19	7	0.5959	0	0.0154
**CARDYYYYGMDVW**	IGHV4-59*01F		IGHJ6*02F	13	8	0.3801	0	0.012
CASYLNWGEDYW	IGHV3-11*03F=IGHV3-11*05F	IGHD7-27*01F	IGHJ4*02F	12	9	0.3387	0	0.0051
CARVELGISPNDAFDIW	IGHV3-7*03F	IGHD7-27*01F	IGHJ3*02F	17	10	0.3011	0	0.0051
**CVHRLSTLRGDIPYFDYW**	IGHV2-70*01F	IGHD3-16*02F	IGHJ4*02F	18	11	0.0376	0	0.0186
**CVHRLSTLRGDIPYFDYW**	IGHV2-5*02F	IGHD3-10*01F	IGHJ4*02F	18	12	0.0314	0	0.0051
**CARDYYYYGMDVW**	IGHV4-61*01F		IGHJ6*02F	13	13	0.026	0	0.0051
CARRSNYASGFDYW	IGHV5-51*01F	IGHD3-10*01F	IGHJ4*02F	14	14	0.0125	0	0.0114
CAKGYSSSWYYFDYW	IGHV3-9*01F	IGHD6-13*01F	IGHJ4*02F	15	15	0.0067	0	0.0051
CVGVRTGTSCYDSW	IGHV4-31*03F=IGHV4-31*04F	IGHD2-2*01F	IGHJ5*01F	14	16	0.0063	0	0.2111
CARSSVHLAFDIW	IGHV2-5*01F	IGHD3-10*02F	IGHJ3*02F	13	17	0.0063	0	0.139
CARDKVAGEGYFHYW	IGHV4-4*02F	IGHD6-19*01F	IGHJ4*02F	15	18	0.0063	0	0.0875
CAKDMWTARSGENYYYYGMDVW	IGHV3-23*01F=IGHV3-23*04F	IGHD3-10*02F	IGHJ6*02F	22	19	0.0063	0	0.0257
CAGGSHPKIVTSLDYW	IGHV3-7*01F	IGHD5-12*01F	IGHJ4*02F	16	20	0.0063	0	0.0103
CARHVMYHSGSYSLHWYFDLW	IGHV5-51*01F	IGHD1-26*01F	IGHJ2*01F	21	21	0.0063	0	0.0051
CARSPVLNHDAFDIW	IGHV5-51*01F	IGHD2-8*01F	IGHJ3*02F	15	22	0.2384	0.0785	0
**% of total repertoire**						**16.3214**		**0.9217**

Clonotypes with the same highlghted background demonstrate VDJ convergent recombination; clonotypes in bold are amonsgt the top 100 most abundant across all developmental stages (Fig 2b)

Supplementary Table 8. Immunogenetic features of shared clonotypes between FBM and postnatal samples

junction	VH	DH	JH	CDR3L	#	FBM, %	cPB, %	aPB, %
CAHRRFLGSTGGFDYW	IGHV2-5*02F	IGHD3-10*02F	IGHJ4*02F	16	1	0.1951	0.0065	0
CARRNLTGDFDYW	IGHV5-51*01F	IGHD7-27*01F	IGHJ4*02F	13	2	0.1192	0.0065	0
CAREVWGDAFDIW	IGHV6-1*01F=IGHV6-1*02F	IGHD3-16*01F	IGHJ3*02F	13	3	0.0975	0.0065	0
CARGTGDWYFDLW	IGHV6-1*01F=IGHV6-1*02F	IGHD7-27*01F	IGHJ2*01F	13	4	0.0975	0.0065	0
CAHRERRFAFDIW	IGHV2-5*02F	IGHD3-3*01F	IGHJ3*02F	13	5	0.0759	0.0065	0
CARDGWDWYFDLW	IGHV6-1*01F=IGHV6-1*02F	IGHD3-10*01F	IGHJ2*01F	13	6	0.0759	0.0065	0
CARGGNWGPPPNDAFDIW	IGHV6-1*01F=IGHV6-1*02F	IGHD7-27*01F	IGHJ3*02F	18	7	0.0759	0.0065	0
CARTPLGKDAFDIW	IGHV2-70*01F	IGHD7-27*01F	IGHJ3*02F	14	8	0.0759	0.0065	0
CARFLTIFGVVIDAFDIW	IGHV6-1*01F=IGHV6-1*02F	IGHD3-3*01F	IGHJ3*02F	18	9	0.0683	0.0109	0
CARLELLGMVEAFDIW	IGHV5-51*01F	IGHD1-20*01F	IGHJ3*02F	16	10	0.0669	0.0121	0
CAHRRPFLWYFDLW	IGHV2-5*02F	IGHD1-14*01ORF	IGHJ2*01F	14	11	0.065	0.0065	0
CARSMARRGSYYFDYW	IGHV2-70*11F	IGHD1-26*01F	IGHJ4*02F	16	12	0.0569	0.0055	0
CARGVAGDAFDIW	IGHV6-1*01F=IGHV6-1*02F	IGHD2-15*01F	IGHJ3*02F	13	13	0.0557	0.006	0
CAREGGNSIIDYW	IGHV6-1*01F=IGHV6-1*02F	IGHD4-23*01ORF	IGHJ4*02F	13	14	0.0455	0.0055	0
CAREVTGDRPGYFDYW	IGHV6-1*01F=IGHV6-1*02F	IGHD7-27*01F	IGHJ4*02F	16	15	0.0455	0.0055	0
CAHENLTGVYYW	IGHV2-5*02F	IGHD7-27*01F	IGHJ4*02F	12	16	0.0446	0.006	0
CARHVGDIW	IGHV5-51*01F	IGHD1-26*01F	IGHJ3*02F	9	17	0.0446	0.006	0
CAREGIAAAGLLDYW	IGHV6-1*01F=IGHV6-1*02F	IGHD6-13*01F	IGHJ4*02F	15	18	0.0341	0.0109	0
CARGSAWGSGDIW	IGHV6-1*01F=IGHV6-1*02F	IGHD3-16*01F	IGHJ3*02F	13	19	0.0341	0.0109	0
CARERGGSELDDAFDIW	IGHV6-1*01F=IGHV6-1*02F	IGHD3-22*01F	IGHJ3*02F	17	20	0.0325	0.0065	0
CARQLLTGVDDWYFDLW	IGHV4-39*01F	IGHD7-27*01F	IGHJ2*01F	17	21	0.0325	0.0065	0
CARGDILTGYSNWFDPW	IGHV3-64*01F	IGHD3-9*01F	IGHJ5*02F	17	22	0.0228	0.0055	0
CARILQSSSWYDAFDIW	IGHV2-70*01F	IGHD6-13*01F	IGHJ3*02F	17	23	0.0228	0.0055	0
CARSIPYRWFRELLYYFDYW	IGHV5-51*01F	IGHD3-10*01F	IGHJ4*02F	20	24	0.0228	0.0055	0
CAHAIRGGGAFDIW	IGHV2-5*02F	IGHD3-16*01F	IGHJ3*02F	14	25	0.0223	0.006	0
CAHKDDGYWYFDLW	IGHV2-5*02F	IGHD3-10*01F	IGHJ2*01F	14	26	0.0223	0.006	0
CARGFNWAAAFDIW	IGHV1-2*02F	IGHD1-1*01F	IGHJ3*02F	14	27	0.0223	0.006	0
CARPLKLYVDAFDIW	IGHV5-51*01F	IGHD3-10*02F	IGHJ3*02F	15	28	0.0223	0.006	0
CARGTGDGDAFDIW	IGHV3-30*01F	IGHD7-27*01F	IGHJ3*02F	14	29	0.0114	0.0109	0
CARDLDWRDAFDIW	IGHV6-1*01F=IGHV6-1*02F	IGHD3-9*01F	IGHJ3*02F	14	30	0.0114	0.0055	0
CARDSPTGDDPLDAFDIW	IGHV4-31*03F=IGHV4-31*04F	IGHD7-27*01F	IGHJ3*02F	18	31	0.0114	0.0055	0
CARGRSTGEDFDYW	IGHV5-51*01F	IGHD7-27*01F	IGHJ4*02F	14	32	0.0114	0.0055	0
CARGTTWYNWNDVYFDYW	IGHV3-53*01F=IGHV3-53*02F	IGHD1-1*01F	IGHJ4*02F	18	33	0.0114	0.0055	0
CARLVVANWGYNWFDPW	IGHV5-51*01F	IGHD7-27*01F	IGHJ5*02F	17	34	0.0114	0.0055	0
CARNTEGGYCSSTSCWYFDLW	IGHV5-51*01F	IGHD2-2*01F	IGHJ2*01F	21	35	0.0114	0.0055	0
CARPGSAGDYW	IGHV5-51*01F	IGHD1-1*01F	IGHJ4*02F	11	36	0.0114	0.0055	0
CARRRGYSSSWYAFDIW	IGHV5-51*01F	IGHD6-13*01F	IGHJ3*02F	17	37	0.0114	0.0055	0
CARRSAAAGIFDYW	IGHV5-51*01F	IGHD6-13*01F	IGHJ4*02F	14	38	0.0114	0.0055	0
CARVGIITIFGVVIRGGEVFDYW	IGHV1-69*02F=IGHV1-69*04F=IGHV1-69*09F	IGHD3-3*01F	IGHJ4*02F	23	39	0.0114	0.0055	0
CAREDAFDIW	IGHV1-2*02F		IGHJ3*02F	10	40	0.0111	0.0483	0
CARVRANWGYFDLW	IGHV6-1*01F=IGHV6-1*02F	IGHD7-27*01F	IGHJ2*01F	14	41	0.0111	0.0325	0
CARARFLEWLFDYW	IGHV5-51*01F	IGHD3-3*01F	IGHJ4*02F	14	42	0.0111	0.006	0
CARDGDLDPFDYW	IGHV1-2*02F	IGHD3-10*01F	IGHJ4*02F	13	43	0.0111	0.006	0
CARDRGRTGDRGDYFDYW	IGHV6-1*01F=IGHV6-1*02F	IGHD7-27*01F	IGHJ4*02F	18	44	0.0111	0.006	0
CARERITIFGVVDYW	IGHV6-1*01F=IGHV6-1*02F	IGHD3-3*01F	IGHJ4*02F	15	45	0.0111	0.006	0
CARFCEGRDWGRGFDYW	IGHV6-1*01F=IGHV6-1*02F	IGHD3-16*01F	IGHJ4*02F	17	46	0.0111	0.006	0
CARFLEWYFDYW	IGHV1-8*01F=IGHV1-8*02F	IGHD3-3*01F	IGHJ4*02F	12	47	0.0111	0.006	0
CARGNRPLAFDIW	IGHV6-1*01F=IGHV6-1*02F	IGHD1-26*01F	IGHJ3*02F	13	48	0.0111	0.006	0
CARGRANWGTYFDYW	IGHV6-1*01F=IGHV6-1*02F	IGHD7-27*01F	IGHJ4*02F	15	49	0.0111	0.006	0
CARRLTGDAFDIW	IGHV1-69*02F=IGHV1-69*04F=IGHV1-69*09F	IGHD7-27*01F	IGHJ3*02F	13	50	0.0111	0.006	0
CARCTRRTPLNDGVDYW	IGHV1-2*02F	IGHD3-10*02F	IGHJ4*02F	17	51	0.0108	0.397	0
CVYDFWSGYPVW	IGHV1-8*01F=IGHV1-8*02F	IGHD3-3*01F	IGHJ4*02F	12	52	0.0108	0.0195	0
CARDLGWYFDLW	IGHV3-7*01F	IGHD5-12*01F	IGHJ2*01F	12	53	0.0108	0.0065	0
CARFGDGDWYFDLW	IGHV4-39*07F	IGHD3-10*01F	IGHJ2*01F	14	54	0.0108	0.0065	0
CARIPRRIDAFDIW	IGHV2-5*02F	IGHD3-22*01F	IGHJ3*02F	14	55	0.0108	0.0065	0
CARRVAGFDYW	IGHV4-59*08F	IGHD6-19*01F	IGHJ4*02F	11	56	0.0108	0.0065	0
CARVLSIAARYYYGMDVW	IGHV6-1*01F=IGHV6-1*02F	IGHD6-6*01F	IGHJ6*02F	18	57	0.0108	0.0065	0
CASLGLGTEEYYFDYW	IGHV1-2*02F	IGHD6-13*01F	IGHJ4*02F	16	58	0.0108	0.0065	0
CASRLGLGIPYWYFDLW	IGHV4-39*01F	IGHD7-27*01F	IGHJ2*01F	17	59	0.0108	0.0065	0
CARYLTGTDAFDIW	IGHV6-1*01F=IGHV6-1*02F	IGHD1-20*01F	IGHJ3*02F	14	60	0.0557	0	0.01
CAREGICDSSWYFQHW	IGHV6-1*01F=IGHV6-1*02F	IGHD6-13*01F	IGHJ1*01F	16	61	0.0433	0	0.0108
CARGYGSGSYEYFQHW	IGHV6-1*01F=IGHV6-1*02F	IGHD3-10*01F	IGHJ1*01F	16	62	0.0341	0	0.01
CAHQELLWFGELFDYW	IGHV2-5*02F	IGHD3-10*01F	IGHJ4*02F	16	63	0.0334	0	0.0104
CALGVVEASGGWYRSGLDPW	IGHV1-2*04F	IGHD2-15*01F	IGHJ5*02F	20	64	0.0223	0	0.0114
CARHKEFNWFDPW	IGHV5-51*01F	IGHD3-9*01F	IGHJ5*02F	13	65	0.0217	0	0.01
CARMGGHAFDIW	IGHV4-31*03F=IGHV4-31*04F	IGHD3-16*01F	IGHJ3*02F	12	66	0.0217	0	0.01
CGRTYNYDTSGWGFEYW	IGHV1-46*01F=IGHV1-46*02F=IGHV1-46*03F	IGHD3-22*01F	IGHJ4*02F	17	67	0.0114	0	0.4102
CARAERYDFWSGYLYYFDYW	IGHV1-8*01F=IGHV1-8*02F	IGHD3-3*01F	IGHJ4*02F	20	68	0.0114	0	0.1823
CARRRAYGGNWDSGTFEIW	IGHV2-5*02F	IGHD2-21*02F	IGHJ3*02F	19	69	0.0114	0	0.1506
CARDSRRWELLLSPGYFDLW	IGHV1-2*02F	IGHD2-15*01F	IGHJ2*01F	20	70	0.0114	0	0.0114
CARERRDAFDIW	IGHV1-46*01F=IGHV1-46*02F=IGHV1-46*03F	IGHD4-23*01ORF	IGHJ3*02F	12	71	0.0114	0	0.0114
CARDRSSSVDYW	IGHV7-4-1*02F	IGHD6-6*01F	IGHJ4*02F	12	72	0.0114	0	0.0108
CAHPSNGDAFDIW	IGHV2-5*02F	IGHD2-8*02F	IGHJ3*02F	13	73	0.0114	0	0.01
CARGWVGDAFDIW	IGHV6-1*01F=IGHV6-1*02F	IGHD6-19*01F	IGHJ3*02F	13	74	0.0114	0	0.01
CARDRWFGELGSFDYW	IGHV6-1*01F=IGHV6-1*02F	IGHD3-10*01F	IGHJ4*02F	16	75	0.0111	0	0.0731
CARAVWGGAVDW	IGHV6-1*01F=IGHV6-1*02F	IGHD3-16*01F	IGHJ4*02F	12	76	0.0111	0	0.0522
CAREDRDAWAVGASYYYYYYMDVW	IGHV6-1*01F=IGHV6-1*02F	IGHD3-10*02F	IGHJ6*03F	24	77	0.0111	0	0.0313
CARKAGDAAGMDVW	IGHV2-70*01F	IGHD7-27*01F	IGHJ6*02F	14	78	0.0111	0	0.0215
CARSYGSGSGWFDPW	IGHV2-70*01F	IGHD3-10*01F	IGHJ5*02F	15	79	0.0111	0	0.02
CARVLYCGGDCSSRYYYGMDVW	IGHV1-2*02F=IGHV1-2*05F	IGHD2-21*01F	IGHJ6*02F	22	80	0.0111	0	0.02
CAHSSITIFGVVIPYNWFDPW	IGHV2-5*01F	IGHD3-3*01F	IGHJ5*02F	21	81	0.0111	0	0.01
CARSEVVGPSGEDFDNW	IGHV6-1*01F=IGHV6-1*02F	IGHD1-26*01F	IGHJ4*02F	17	82	0.0108	0	0.03
CARVDYGLHAFDIW	IGHV4-59*01F	IGHD3-16*01F	IGHJ3*02F	14	83	0.0108	0	0.01
**% total repertoire**						**2.3641**	**0.854**	**1.1374**

Supplementary Table 9. Immunogenetic features of clonotypes belonging to CLL stereotypic IgH receptors

'confidence' is based on the difference between the assignment score for that sequence and the minimum assignment score for that subset.

sample	CLL subset	confidence	score	VH	JH	CDR3len	mut	junction	frame
FL	CLL#5	average	27.5457	IGHV7-4-1*02	IGHJ6*02	20	unmutated	CARGGITGANSKYYYYYGMDVW	in-frame
FL	CLL#5	borderline	22.7507	IGHV1-69*13	IGHJ6*02	20	unmutated	CARDHPSGDGAYYYYYYGMDVW	in-frame
FL	CLL#5	borderline	21.5542	IGHV1-69*13	IGHJ6*02	20	unmutated	CARTSKDIVATIRYYYYGMDVW	in-frame
FBM	CLL#5	average	28.1072	IGHV1-69*01=IGHV1-69*12=IGHV1-69*13	IGHJ6*03	20	unmutated	CARDGVRDQTGFYYYYYYMDVW	in-frame
FBM	CLL#5	borderline	21.7374	IGHV1-46*01=IGHV1-46*02=IGHV1-46*03	IGHJ6*03	20	unmutated	CARVRLGPVVAGYYYYYYMDVW	in-frame
FBM	CLL#5	borderline	22.1194	IGHV1-2*02=IGHV1-2*03=IGHV1-2*04	IGHJ6*02	20	unmutated	CARGGREQLVRYYYYYYGMDVW	in-frame
FBM	CLL#5	high	35.2981	IGHV5-51*01	IGHJ6*03	20	unmutated	CARLTIFGVVIIDYYYYYMDVW	in-frame
cPB	CLL#5	average	31.8392	IGHV1-8*02	IGHJ6*03	20	unmutated	CARAGRPVVIIIPYYYYYMDVW	in-frame
cPB	CLL#5	borderline	21.5374	IGHV1-8*01=IGHV1-8*02	IGHJ6*03	20	unmutated	CARGWTRITIFGVVTYYYMDVW	in-frame
cPB	CLL#5	borderline	21.0583	IGHV1-69*01=IGHV1-69*12=IGHV1-69*13	IGHJ6*03	20	unmutated	CARGAGIAVAGTRNYYYYMDVW	in-frame
cPB	CLL#5	high	34.6449	IGHV1-69*13	IGHJ6*03	20	unmutated	CARDEVVAVAPRHYYYYYMDVW	in-frame
cPB	CLL#5	low	23.9373	IGHV1-18*01=IGHV1-18*04	IGHJ6*03	20	unmutated	CARDPVTTVTTQDYYYYYMDVW	in-frame
cPB	CLL#5	average	27.1534	IGHV1-69*01=IGHV1-69*12=IGHV1-69*13	IGHJ6*02	20	unmutated	CARNPGIAVVPAQYYCYGMDVW	in-frame
cPB	CLL#5	low	25.2939	IGHV1-3*01	IGHJ6*02	20	unmutated	CARDSVDIVATWNYYYYGMDVW	in-frame
cPB	CLL#5	low	23.9709	IGHV5-10-1*01=IGHV5-10-1*03	IGHJ6*02	20	unmutated	CARQDQIAVAGTVYYYYGMDVW	in-frame
cPB	CLL#5	low	23.8836	IGHV1-69*13	IGHJ6*02	20	unmutated	CASPTGSGSYYTYYYYYGMDVW	in-frame
cPB	CLL#5	average	32.7617	IGHV1-18*01=IGHV1-18*04	IGHJ6*03	20	unmutated	CARVVVAATIEDYYYYYYMDVW	in-frame
cPB	CLL#5	average	29.2986	IGHV1-46*01=IGHV1-46*03	IGHJ6*03	20	unmutated	CARDKGVGVNLNYYYYYYMDVW	in-frame
cPB	CLL#5	borderline	22.0674	IGHV1-2*02	IGHJ6*03	20	unmutated	CARVGMGVVAGTTYYYYYMDVW	in-frame
cPB	CLL#5	borderline	21.9354	IGHV5-10-1*01=IGHV5-10-1*03	IGHJ6*03	20	unmutated	CARHGNEGIAARPYYYYYMDVW	in-frame
cPB	CLL#5	borderline	21.493	IGHV1-69*05=IGHV1-69*13	IGHJ6*03	20	unmutated	CARDLRRDGYNDYYYYYYMDVW	in-frame
cPB	CLL#5	low	25.0688	IGHV1-2*02	IGHJ6*03	20	unmutated	CARASISPVDGGYYYYYYMDVW	in-frame
cPB	CLL#5	low	23.6375	IGHV1-8*01=IGHV1-8*02	IGHJ6*03	20	unmutated	CARGMTMVTTLMYYYYYYMDVW	in-frame
cPB	CLL#5	average	27.7249	IGHV1-18*01	IGHJ6*02	20	unmutated	CARDTPPGVVPAAPYYYGMDVW	in-frame
cPB	CLL#5	average	31.9444	IGHV1-18*01=IGHV1-18*04	IGHJ6*02	20	unmutated	CARVDIVVVVAALYYYYGMDVW	in-frame
cPB	CLL#5	borderline	21.2668	IGHV1-69*02=IGHV1-69*04=IGHV1-69*09	IGHJ6*02	20	unmutated	CARDPLTVAATNYYYYYGMDVW	in-frame
cPB	CLL#5	borderline	22.5637	IGHV1-69*01=IGHV1-69*12=IGHV1-69*13	IGHJ6*02	20	unmutated	CARGQGRGWFRESHYYYGMDVW	in-frame
cPB	CLL#5	borderline	21.0238	IGHV5-51*01	IGHJ6*02	20	unmutated	CARQGITMVQGANYYYYGMDVW	in-frame
cPB	CLL#5	borderline	22.5168	IGHV1-69*01=IGHV1-69*12=IGHV1-69*13	IGHJ6*03	20	unmutated	CARDGGTISYPNYYYYYYMDVW	in-frame
cPB	CLL#5	borderline	21.0149	IGHV1-69*01=IGHV1-69*12=IGHV1-69*13	IGHJ6*01	20	unmutated	CARALNTAMVSPYYYYYGMDVW	in-frame
cPB	CLL#5	high	37.2933	IGHV5-10-1*01=IGHV5-10-1*03	IGHJ6*03	20	unmutated	CARRAIFGVVNSYYYYYYMDVW	in-frame
aPB	CLL#5	average	30.7116	IGHV1-2*02	IGHJ6*03	20	unmutated	CARDRVTMVQANYYYYYYMDVW	in-frame
aPB	CLL#5	average	27.5136	IGHV1-69*02=IGHV1-69*04=IGHV1-69*09	IGHJ6*02	20	unmutated	CARESEAVVVPAAMYYYGMDVW	in-frame
aPB	CLL#5	average	28.5702	IGHV1-69*13	IGHJ6*02	20	unmutated	CAREDLFMVQGNYYYYYGMDVW	in-frame
aPB	CLL#5	average	26.7655	IGHV1-46*01=IGHV1-46*03	IGHJ6*02	20	unmutated	CARDLIVVVVAATYYYYGMDVW	in-frame
aPB	CLL#5	average	26.6604	IGHV1-8*01=IGHV1-8*02	IGHJ6*02	20	unmutated	CARGDIVVVPGAHYYYYGMDVW	in-frame
aPB	CLL#5	borderline	22.9105	IGHV1-2*05	IGHJ6*02	20	unmutated	CARGGITGTTGTYHYYYGMDVW	in-frame
aPB	CLL#5	borderline	21.5591	IGHV1-8*01	IGHJ6*02	20	unmutated	CARGGIVGATVDYYYYYGMDVW	in-frame
aPB	CLL#5	low	24.7688	IGHV1-8*01=IGHV1-8*02	IGHJ6*02	20	unmutated	CARGPSSGTVTTFDYYYGMDVW	in-frame
aPB	CLL#5	low	24.9821	IGHV5-10-1*01=IGHV5-10-1*03	IGHJ6*02	20	unmutated	CARHEEVPVAGTVYYYYGMDVW	in-frame
FL	CLL#28A	average	48.0086	IGHV7-4-1*02	IGHJ6*02	17	unmutated	**CASEGSGSYYYYYYGMDVW**	in-frame
FL	CLL#28A	average	45.6756	IGHV1-24*01	IGHJ6*02	17	unmutated	**CASEGSGSYYYYYYGMDVW**	in-frame
FL	CLL#28A	high	55.646	IGHV1-2*02	IGHJ6*02	17	unmutated	CARDYSGSYYYYYYGMDVW	in-frame
FBM	CLL#28A	average	46.8734	IGHV1-2*02	IGHJ6*02	17	unmutated	CARTDSSSSYYYYYGMDVW	in-frame
FBM	CLL#28A	high	50.2292	IGHV1-2*02	IGHJ6*02	17	unmutated	CARAYSSSFYYYYYGMDVW	in-frame
cPB	CLL#28A	average	45.7733	IGHV1-2*02	IGHJ6*03	17	unmutated	CARGASGSYYYYYYYMDVW	in-frame
FL	CLL#1	average	22.3217	IGHV1-18*01	IGHJ4*02	13	unmutated	CARAQALGIRPLDYW	in-frame
FL	CLL#1	low	13.4818	IGHV1-2*03	IGHJ4*02	13	unmutated	CARDGRLVLNPFDYW	in-frame
FL	CLL#1	borderline	8.671	IGHV1-2*02	IGHJ4*02	13	unmutated	CARVSFLIEGYFDYW	in-frame
FL	CLL#1	average	28.1086	IGHV1-2*02	IGHJ1*01	13	unmutated	CASEQWLVPQYFQHW	in-frame
FL	CLL#1	borderline	7.8789	IGHV1-2*02	IGHJ4*01	13	unmutated	CAREGKLGTPYLTTW	in-frame
FL	CLL#1	borderline	7.8832	IGHV1-2*02	IGHJ4*02	13	unmutated	CARPQLGVPYYFDYW	in-frame
FL	CLL#1	average	17.2339	IGHV1-8*02	IGHJ4*02	13	unmutated	CARAQALGIGPLDYW	in-frame
FL	CLL#1	borderline	8.3402	IGHV1-2*02	IGHJ4*02	13	unmutated	CARVPRLGLPFFDYW	in-frame
FBM	CLL#1	average	24.2051	IGHV1-2*02	IGHJ4*02	13	unmutated	CAIPQQLVPETFDYW	in-frame
FBM	CLL#1	borderline	7.3298	IGHV1-2*02	IGHJ2*01	13	unmutated	CAREARLV**YFDLW	in-frame
FBM	CLL#1	borderline	6.6296	IGHV1-46*01=IGHV1-46*02=IGHV1-46*03	IGHJ4*02	13	unmutated	CARVQRGVLHYFGYW	in-frame
FBM	CLL#1	borderline	7.5937	IGHV1-2*04	IGHJ4*02	13	unmutated	CARAQTAVQGVIDYW	in-frame
FBM	CLL#1	low	13.2084	IGHV1-8*01=IGHV1-8*02	IGHJ4*02	13	unmutated	CARELWFRELYFDYW	in-frame
FBM	CLL#1	low	11.6549	IGHV5-51*01	IGHJ4*02	13	unmutated	CARLSILGMPYFDYW	in-frame
FBM	CLL#1	low	12.9754	IGHV1-8*01=IGHV1-8*02	IGHJ4*02	13	unmutated	CARWSLLGIRHFDYW	in-frame
cPB	CLL#1	average	19.1373	IGHV1-46*01=IGHV1-46*02=IGHV1-46*03	IGHJ4*02	13	unmutated	CARDGWLVNGSCDYW	in-frame
cPB	CLL#1	borderline	6.4035	IGHV1-3*01	IGHJ4*02	13	unmutated	CARAGKLEQGLFDYW	in-frame
cPB	CLL#1	low	12.7636	IGHV1-8*01=IGHV1-8*02	IGHJ4*02	13	unmutated	CARGQIAVAGNFDYW	in-frame
cPB	CLL#1	low	11.7876	IGHV1-18*01=IGHV1-18*04	IGHJ1*01	13	unmutated	CARRQQLPAEYFQHW	in-frame
cPB	CLL#1	low	10.7205	IGHV1-18*01=IGHV1-18*04	IGHJ4*02	13	unmutated	CARGHQWELPHFDYW	in-frame
cPB	CLL#1	average	17.6761	IGHV5-10-1*01=IGHV5-10-1*03	IGHJ4*02	13	unmutated	**CARRGAMVPRSFDYW**	in-frame
cPB	CLL#1	borderline	6.4664	IGHV5-51*01	IGHJ4*02	13	unmutated	**CARRGAMVPRSFDYW**	in-frame
cPB	CLL#1	borderline	5.5759	IGHV1-2*02	IGHJ4*02	13	unmutated	CARGHWVVVTPVDYW	in-frame
cPB	CLL#1	borderline	4.6186	IGHV1-8*01=IGHV1-8*02	IGHJ4*02	13	unmutated	CARRNQWLVRNFDYW	in-frame
cPB	CLL#1	average	14.4517	IGHV1-3*01	IGHJ4*02	13	unmutated	CARSEWELLLFFDYW	in-frame
cPB	CLL#1	average	18.0163	IGHV1-3*01	IGHJ4*02	13	unmutated	CARGSELGVRYFDYW	in-frame
cPB	CLL#1	average	17.9589	IGHV1-18*01	IGHJ4*02	13	unmutated	CARGGWVVVAPFDYW	in-frame
cPB	CLL#1	borderline	8.3642	IGHV1-3*01	IGHJ4*02	13	unmutated	CARAEWIAVWYFDYW	in-frame
cPB	CLL#1	borderline	6.7906	IGHV1-18*01=IGHV1-18*04	IGHJ4*02	13	unmutated	CARDRWFLFSHIDYW	in-frame
cPB	CLL#1	borderline	6.4606	IGHV1-3*01	IGHJ4*02	13	unmutated	CARDQEVVAAPPDYW	in-frame
cPB	CLL#1	high	39.3958	IGHV1-46*01=IGHV1-46*03	IGHJ4*02	13	unmutated	CARGQWLVDSYFDYW	in-frame
cPB	CLL#1	low	11.1913	IGHV1-3*01	IGHJ4*02	13	unmutated	CARVSVVVAYYFDYW	in-frame
cPB	CLL#1	low	10.7715	IGHV1-18*01=IGHV1-18*04	IGHJ4*02	13	unmutated	CAREQVAATSTLDYW	in-frame
cPB	CLL#1	low	10.3091	IGHV1-18*01	IGHJ4*02	13	unmutated	CARESRVVQGNFDYW	in-frame
cPB	CLL#1	low	10.4006	IGHV1-18*01=IGHV1-18*04	IGHJ4*02	13	unmutated	CARDQAVAGTSLDYW	in-frame
cPB	CLL#1	low	9.4747	IGHV1-2*02	IGHJ4*02	13	unmutated	CAREQDNGPYYFDYW	in-frame
aPB	CLL#1	average	27.7989	IGHV1-3*01	IGHJ4*02=IGHJ5*02	13	unmutated	CARTQWLVTGTVENW	in-frame
aPB	CLL#1	average	28.662	IGHV1-8*01=IGHV1-8*02	IGHJ4*02	13	unmutated	CARGQQLVGASFDYW	in-frame
aPB	CLL#1	average	19.69	IGHV1-18*01	IGHJ4*02	13	unmutated	CARSWQLVPSYFDYW	in-frame
aPB	CLL#1	borderline	7.0919	IGHV1-3*01	IGHJ4*02	13	unmutated	CAREGAVGATWFDYW	in-frame
aPB	CLL#1	high	35.9479	IGHV1-3*01	IGHJ4*02=IGHJ5*02	13	unmutated	CARTQWLVTGTVVYW	in-frame
aPB	CLL#1	high	35.6416	IGHV1-3*01	IGHJ4*02	13	unmutated	CARTQWLVTGTVEYW	in-frame
aPB	CLL#1	high	29.7688	IGHV1-3*01	IGHJ4*02	13	unmutated	CARGQWELLDPFDYW	in-frame

Sequences in bold demonstrate convergent recombinations
