# Supplementary material for: High resolution IgH repertoire analysis reveals fetal liver as the likely origin of life-long, innate B lymphopoiesis in humans
Source: Clin Immunol. 2017 Oct;183:8–16. doi: 10.1016/j.clim.2017.06.005 (PMC5678457; doi:10.1016/j.clim.2017.06.005)
Supplement: Fig. S1 [file mmc2.pdf]

Figure S1

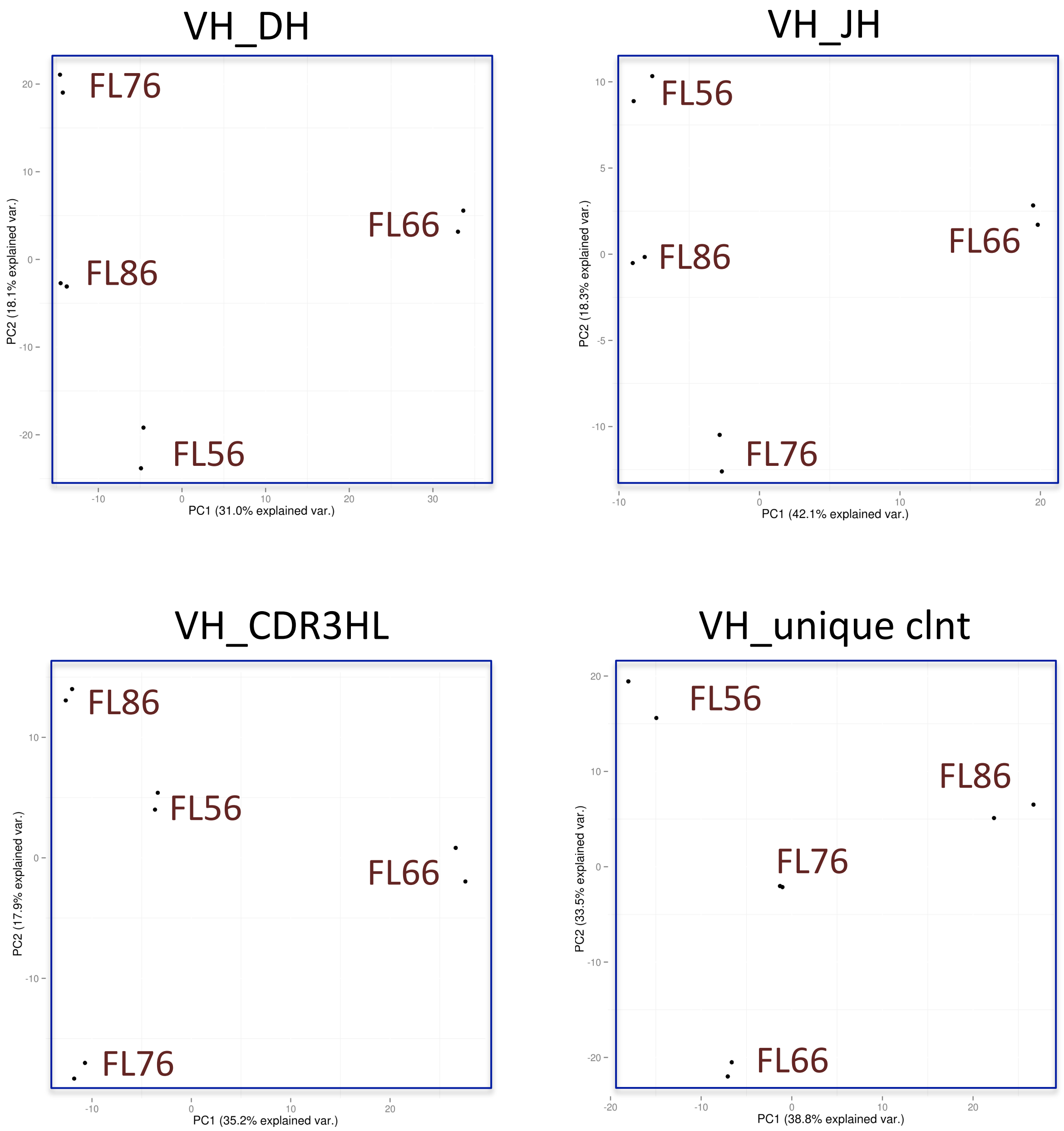

Supplementary Figure 1. Principal component analysis of immunogenetic feature distributions in duplicate FL B cell samples. VH-DH, VH-JH, VH-CDR3L, and VH, all counted in unique clonotypes (clnt).
