# Supplementary material for: High resolution IgH repertoire analysis reveals fetal liver as the likely origin of life-long, innate B lymphopoiesis in humans
Source: Clin Immunol. 2017 Oct;183:8–16. doi: 10.1016/j.clim.2017.06.005 (PMC5678457; doi:10.1016/j.clim.2017.06.005)
Supplement: Supplementary file 3 — Supplementary methods. [file mmc3.docx]

**Supplementary methods**

**Flow cytometric analysis and sorting**

Cells were processed and stained for flow cytometry with up to eight fluorophore-conjugated monoclonal antibody (mAb) as previously described ^1^. Antibodies used: viability efluor506 (eBioscience), CD34 PECy7 (8G12: BD), CD34 APC eFluor780 (8G12: eBioscience), CD19 APC (HIB19: BD/Biolegend), CD19 Alexafluor 700 (HIB-19: eBioscience), CD10 PE/ PE Cy7 (eBioCB-CALLA: eBioscience), CD27 PE/eFluor450 (O323: ebioscience), CD27 PerCP-Cy5.5 (M-T271: Biolegend), IgM FITC/ PerCP-Cy5.5 (MHM-88: Biolegend), IgD FITC/PerCP Cy5.5 (IA6-2: Biolegend), IgD (IADB6: Beckman Coulter). Samples were analysed using a BD LSR Fortessa or FACSAria II (Becton Dickinson). Bulk CD34-CD19+ B cells were sorted for BCR repertoire analysis, however their composition was determined by immunophenotypic definitions of B cell subsets (Pre B, immature, transitional and naïve B cells) according to previously published reports (data from human fetal B cell published literature was used where available to define these populations)^2-8^. Gates were set with unstained and fluorescence minus one controls, on viable cells identified using DAPI or viability eFluor506. Data were analysed on FlowJo software (Tree Star). Gating strategies are as described in the results.

**Spectratyping**

Total RNA from up to 500,000 cells was isolated with the RNeasy plus micro kit (Qiagen, Hilden, Germany) and reverse transcribed by random priming using the RevertAid™ first strand cDNA synthesis kit (Fermentas, York, UK).

For IgM heavy chain CDR3 size spectratyping, V_H_1-6-specific transcripts were amplified by independent polymerase chain reactions (PCR) using specific primers according to a protocol described previously ^9^.

The following general PCR protocol was used: cDNA, forward primer 0.5µM, reverse primer 0.5µM, dNTPs 0.2mM, 10x DreamTaq™ buffer (Fermentas) 5µL, DreamTaq™ polymerase (Fermentas) 1.2U, ddH_2_O up to 50µL. Amplification was performed in a 2720 thermal cycler (Applied Biosystems, Carlsbad, USA) with an initial denaturation step of 95°C for 5min followed by 30 cycles at 95°C for 30sec, 66°C for 30sec, 72°C for 1min. A final elongation step of 72°C for 10min terminated the reaction. 20µL of the PCR product were visualised on a 1% agarose gel, and PCR products of the expected size (≈600bp) were subjected to a run-off reaction with a single fluorescent primer using the following protocol and conditions: PCR product 3µL, Cµ run-off primer 0.5µM, dNTPs, 0.2mM, 10x DreamTaq™ buffer (Fermentas) 2µL, DMSO (Sigma, St. Louis, USA) 1µL, DreamTaq™ polymerase (Fermentas) 0.2U, ddH_2_O up to 20µL. Amplification x10 cycles was as above. The primer sequences for the amplification of V_H_1-6 IgM heavy chain families and the final run-off reaction were as follows. Forward primer sequence (5' to 3'); VH1L: CAC ACC ATG GAC TGG ACC TGG AG; VH2L: ATG GAC ATA CTT TGT TCC AGG CTC; VH3L: CCA TGG AGT TGG GCT GAG GTG G; VH4L: ACA TGA AAC AYC TGT BGG TTC TTC C; VH5L: ATG GGG TCA ACC GCC ATC CTC G; VH6L: ATG TCT GTC TCC TTC CTC ATC TTC; Reverse primer sequence (5' to 3'): IgM (Cµ)TGC TGA TGT CAG AGT TGT TCT TGT ATT TC; Run-off primer sequence (5' to 3'): Cµ run-off HEX - AAG GGT TGG GGC GGA TGC.

To visualise the CDR3 length distributions 2μL of the run-off product were mixed with 7.9μL hi-di™ formamide and 0.1µL of GeneScan™-500 LIZ^®^ size standard and separated and analysed in an ABI PRISM 3130 gene analyzer using the gene mapper^®^ software v4.0 (all from Applied Biosystems).

**BCR repertoire analysis by 454 sequencing**

RNA from CD34-CD19+ B cells was extracted with the NucleoSpin RNA XS kit (Macherey-Nagel) according to the manufacturer’s instructions. Reverse transcription using random priming was performed with the RevertAid First Strand cDNA Synthesis kit (Thermo Scientific, Leicestershire, UK). IgH V_H_1-7 families were PCR amplified using specific primers for V_H_1-6 FR1 and V_H_7 FR2 against a common Cµ consensus primer as listed above. In a second, semi-nested PCR GS junior MID sequences were added using the same VH1-7 primers against a different Cµ primer. In both steps of PCR amplification template-free controls were run to exclude contamination. To further minimise risk of contamination, PCR amplifications and library preparations we performed in 5 independent experiments.

PCR products were visualised on a 1% agarose gel and specific bands (approx. 450bp) were cut out and gel purified using the GeneJet gel extraction kit (Thermo Scientific) as per manufacturer’s instructions. Library quantification, pooling and processing were performed following the instructions of the emPCR library A kit (Roche, Basel, Switzerland) and the pooled libraries were sequenced on the GS junior platform (Roche) using the full amplicon processing mode.

In total, to mitigate against cross sample contamination, 20 NGS libraries were prepared and sequenced in 5 independent experiments. Four FL samples were analysed in duplicate assays in 2 independent experiments.

**Bioinformatics**

In the majority of cases, we report a larger number of clonotypes (as defined below) than expected from the estimated number of cells analysed (**Supplementary Table 1**), at least partly representing PCR and sequencing ‘noise’. We opted not to remove this ‘noise’ directly, since statistical error correction and even marginal abundance filtering in samples with low amount of starting material and relatively low read count can have a detrimental effect in the variability and information content of the data. Instead, to minimise such inherent difficulties in our analysis and data, only duplicate libraries or the most abundant clonotypes were used when appropriate.

When IMGT/V-QUEST was used, e.g. for Supplementary Tables 4 and 5, the “Search for insertions and deletions in V-REGION” option was used with otherwise default settings. Rearrangements with identical junctional amino acid sequences and identical V(D)J genes and alleles were defined as clonotypes, based on ^10^. For certain analyses, abundances of features were counted in (unique) clonotypes rather than in read counts to alleviate the effects of technical or biological biases.

To investigate convergent recombinations across samples and developmental stages, we clustered all nucleotide junction sequences per sample allowing for a single difference to the most popular (in reads) sequence, and chose the most popular amino acid junction sequence per cluster to represent all member sequences. All unique amino acid junction sequences per sample constituted the 100% for that sample. We counted how many different IGHV genes were recombined with each amino acid junction sequence, per sample, and then how many junctions had each such count. In a convergent evolution analysis approach where a PCR hybrid can potentially create a clonotype with the same junction but a different V region, signs of PCR hybrid or chimeric sequences would include a visual criss-crossing of differences to germline sequences between the two clonotypes. No such events were observed in the datasets analysed.

**Statistical analysis**

Statistical analysis and data visualisation was performed using the GraphPad statistical package. Mann Witney or Wilcoxon sign rank test were used for unpaired and paired data comparisons, respectively. For multiple groups Kruskal-Wallis with Dunn’s correction or 2-way ANOVA were employed for multiple groups and correction for multiple comparisons was applied as appropriate. Differences in CDR3 length were assessed by the permutational multivariate analysis of variance test. Statistical significance was set at p<0.05.

References

1. Roy A, Cowan G, Mead AJ, et al. Perturbation of fetal liver hematopoietic stem and progenitor cell development by trisomy 21. Proc Natl Acad Sci U S A 2012;109:17579-84.

2. Rother MB, Jensen K, van der Burg M, et al. Decreased IL7Ralpha and TdT expression underlie the skewed immunoglobulin repertoire of human B-cell precursors from fetal origin. Sci Rep 2016;6:33924.

3. McWilliams L, Su KY, Liang X, et al. The human fetal lymphocyte lineage: identification by CD27 and LIN28B expression in B cell progenitors. J Leukoc Biol 2013;94:991-1001.

4. van Zelm MC, van der Burg M, de Ridder D, et al. Ig gene rearrangement steps are initiated in early human precursor B cell subsets and correlate with specific transcription factor expression. J Immunol 2005;175:5912-22.

5. Perez-Andres M, Paiva B, Nieto WG, et al. Human peripheral blood B-cell compartments: a crossroad in B-cell traffic. Cytometry B Clin Cytom 2010;78 Suppl 1:S47-60.

6. Agrawal S, Smith SA, Tangye SG, Sewell WA. Transitional B cell subsets in human bone marrow. Clin Exp Immunol 2013;174:53-9.

7. Bendall SC, Davis KL, Amir el AD, et al. Single-cell trajectory detection uncovers progression and regulatory coordination in human B cell development. Cell 2014;157:714-25.

8. LeBien TW. Fates of human B-cell precursors. Blood 2000;96:9-23.

9. Weller S, Mamani-Matsuda M, Picard C, et al. Somatic diversification in the absence of antigen-driven responses is the hallmark of the IgM+ IgD+ CD27+ B cell repertoire in infants. J ExpMed 2008;205:1331-42.

10. Li S, Lefranc MP, Miles JJ, et al. IMGT/HighV QUEST paradigm for T cell receptor IMGT clonotype diversity and next generation repertoire immunoprofiling. Nat Commun 2013;4:2333.

**Supplementary Tables legends**

**Table S1. Samples studied and their NGS output**

Details of prenatal and postnatal samples used for analysis (fetal liver, FL, n=5; fetal bone marrow, FBM, n=3; child peripheral blood, cPB, n=3, adult peripheral blood, aPB, n=5) and their NGS output showing total read counts; and number of unique and productive clonotypes per sample.

**Table S2. Significance of frequency variability usage of VH genes amongst unique clonotypes**

IGHV1-IGHV7 families were used at varying and often significantly different frequencies in all 4 developmental stages as shown. *p<0.05; **p<0.01; ***p<0.001; ****p<0.0001

**Table S3. Frequency of clonotypes with convergent recombination in FL duplicate libraries**

Analysis of duplicate FL libraries showed evidence of distinct VDJ rearrangements encoding identical CDR3 peptide regions (unique convergent clonotypes). % of convergent clonotypes per sample is depicted in the table.

**Table S4. Representative examples of convergent VDJ recombination between duplicate FL libraries**

Examples of convergent VDJ recombination demonstrating identical CDR3 regions both at the aa and nucleotide level. * Differences in VDJ annotation within (near-) identical nucleotide sequences are due to IMGT/V-QUEST's differential behaviour in accepted mutations depending on VH mutational status

**Table S5. Representative examples of convergent recombination between duplicate FL libraries**

Arbitrarily selected examples of pairs of clonotypes showing evidence of convergent evolution from Suppl Table 4 (coloured the same) were analysed with IMGT/V-QUEST. The full VH alignments of the two assigned VH genes and alleles are shown for either clonotype (#1 and #2).

**Table S6. Immunogenetic features of shared clonotypes between FL and FBM**

Table depicting the 15 clonotypes that were shared between FL and FBM (expressed as 0.37% and 0.22% of reads respectively)

**Table S7. Immunogenetic features of shared clonotypes between FL and postnatal samples**

Table depicting the 22 clonotypes that were shared between FL and aPB (expressed as 16% and 0.92% of reads respectively). Clonotypes with the same highlighted background demonstrate VDJ convergent recombination; clonotypes in bold are amongst the top 100 most abundant across all developmental stages.

**Table S8. Immunogenetic features of shared clonotypes between FBM and postnatal samples**

Table depicting the 83 clonotypes that were shared between FBM and cPB /aPB (expressed as 2.3%, 0.85% and 1.13% of reads respectively).

**Table S9. Immunogenetic features of clonotypes belonging to CLL stereotypic IgH receptors**

Evidence of stereotypic IgH receptors corresponding to 16 major CLL subsets was seen in 3/5 FL B-cell samples and in all FBM and postnatal samples, with the most prevalent CLL#1, CLL#5, and CLL#28A depicted in this table. Sequences in bold demonstrate convergent recombinations. 'Confidence' is based on the difference between the assignment score for that sequence and the minimum assignment score for that subset. For more details please see Bystry et al, 2015.
